# Study on the Potential Mechanism of Tonifying Kidney and Removing Dampness Formula in the Treatment of Postmenopausal Dyslipidemia Based on Network Pharmacology, Molecular Docking and Experimental Evidence

**DOI:** 10.3389/fendo.2022.918469

**Published:** 2022-07-07

**Authors:** Xuewen Li, Hongyan Chen, Hongyan Yang, Jian Liu, Yang Li, Yue Dang, Jiajing Wang, Lei Wang, Jun Li, Guangning Nie

**Affiliations:** ^1^ State Key Laboratory of Dampness Syndrome of Chinese Medicine, The Second Affiliated Hospital of Guangzhou University of Chinese Medicine, Guangzhou, China; ^2^ Department of Cardiovascular Medicine, The Second Affiliated Hospital of Guangzhou University of Chinese Medicine, Guangzhou, China; ^3^ The Second Clinical College of Guangzhou University of Chinese Medicine, Guangzhou University of Chinese Medicine, Guangzhou, China; ^4^ Department of Gynecology, The Second Affiliated Hospital of Guangzhou University of Chinese Medicine, Guangzhou, China; ^5^ College of Traditional Chinese Medicine, Shenyang Pharmaceutical University, Shenyang, China

**Keywords:** TCM, postmenopausal dyslipidemia, tonifying kidney and removing dampness formula, network pharmacology, molecular docking, pharmacological mechanisms

## Abstract

**Background:**

Management of menopausal dyslipidemia is the main measure to reduce the incidence of cardiovascular disease in postmenopausal women. Tonifying Kidney and Removing Dampness Formula (TKRDF) is a traditional Chinese medicine (TCM) formula that ameliorates dyslipidemia in postmenopausal women. This study applied network pharmacology, molecular docking, and *in vitro* and *in vitro* experiments to investigate the underlying mechanism of TKRDF against postmenopausal dyslipidemia.

**Methods:**

Network pharmacology research was first conducted, and the active compounds and targets of TKRDF, as well as the targets of postmenopausal dyslipidemia, were extracted from public databases. Protein–protein interaction (PPI), Gene Ontology (GO), and Kyoto Encyclopedia of Genes and Genomes (KEGG) pathway analysis were used to identify the potential targets and signaling pathways of TKRDF in postmenopausal dyslipidemia. Molecular docking was then performed to evaluate the combination of active compounds with principal targets. Finally, an ovariectomized rat model was used for the *in vivo* experiment and alpha mouse liver 12 (AML12) cells treated with palmitic acid were used for the *in vitro* experiments to provide further evidence for the research.

**Results:**

Based on network pharmacology analysis, we obtained 78 active compounds from TKRDF that acted on 222 targets of postmenopausal dyslipidemia. The analysis results indicated that IL6, TNF, VEGFA, AKT1, MAPK3, MAPK1, PPARG and PIK3CA, etc., were the potentially key targets, and the PI3K/AKT signaling pathway was the possibly crucial pathway for TKRDF to treat postmenopausal dyslipidemia. Molecular docking suggested that the active compounds have good binding activity with the core targets. The *in vivo* and *in vitro* experiments demonstrated that TKRDF ameliorates postmenopausal dyslipidemia by regulating hormone levels, inhibiting inflammation, promoting angiogenesis and inhibiting lipid synthesis, which appear to be related to TKRDF’s regulation of the ERK1/2 and PI3K/AKT signaling pathways.

**Conclusion:**

This study clarified the active ingredients, potential targets, and molecular mechanisms of TKRDF for treating postmenopausal dyslipidemia. It also provided a feasible method to uncover the scientific basis and therapeutic mechanism for prescribing TCM in the treatment of diseases.

## 1 Introduction

Dyslipidemia is a major risk factor for the development of atherosclerotic coronary heart disease (CHD) (1). Premenopausal women develop atherosclerotic CHD relatively rarely compared with men, which is mainly due to the protective effect of estrogen on the cardiovascular system (2). During menopause, estrogen deficiency leads to lipid metabolism disorders, and the prevalence of dyslipidemia also increases, which greatly increases the risk of CHD in postmenopausal women (3). Early treatment of postmenopausal dyslipidemia is important to reduce or postpone the onset of CHD. Estrogen-based menopausal hormone therapy (MHT) is the most common treatment to alleviate menopausal symptoms. However, estrogen use is associated with increased risks of breast cancer and coronary heart disease in older women (aged > 60 years) (4, 5). In 2020, the European Menopause and Andropause Society (EMAS) clearly indicated that MHT is not recommended for the first-line treatment of menopausal women with dyslipidemia (6). In the same year, the American Heart Association (AHA) also pointed out that the current lipid-lowering interventions used in menopausal women still have uncertain data on cardiovascular disease prevention, and further research is needed to formulate evidence-based recommendations specifically for women (7). As a result, a safer and more effective therapy for postmenopausal dyslipidemia is urgently needed.

Traditional Chinese medicine (TCM) is used to treat menopausal symptoms among women in most Asian countries, including China, Japan, South Korea, and Vietnam (8). The basic theory of TCM categorizes menopause as an imbalance of Yin and Yang caused by kidney deficiency. Therefore, the treatment with Chinese patent medicine or Chinese herbal medicine aims to invigorate kidney function and balance Yin and Yang (8, 9). Tonifying Kidney and Removing Dampness Formula (TKRDF) is a classical TCM formula, consisting of Duzhong (Eucommia ulmoides Oliv., bark, dried), Nvzhenzi (Ligustrum lucidum W.T.Aiton, fructus, dried), Baizhu (Atractylodes macrocephala Koidz., rhizome, dried), Chenpi (Citrus aurantium L., pericarpium, dried), Zexie (Alisma plantago-aquatica subsp. orientale (Sam.) Sam., tuber, dried), Gegen (Pueraria montana var. lobata (Willd.) Maesen & S.M.Almeida ex Sanjappa & Predeep, root, dried), Danshen (Salvia miltiorrhiza Bunge, root and rhizoma, drized), and Shanzha (Crataegus pinnatifida Bunge, fructus, dried). It is used extensively in clinical practice. A previous study indicated that TKRDF granules could improve the lipid profile and reduce the related metabolic abnormalities in postmenopausal women with mild dyslipidemia when combined with lifestyle changes (10). However, the therapeutic mechanism by which TKRDF improves postmenopausal dyslipidemia is not yet clear. TCM is characterized by multi-components, multi-targets, and multi-pathways (11, 12). It is difficult to clarify the mechanism by using only traditional pharmacological methods. It is therefore necessary to find and implement the new methods for studying the pharmacological mechanisms of TCM.

Network pharmacology is a novel method based on systems biology principles, constructing and visualizing a ‘multi-gene, multi-target, multi-pathway’ interaction network to evaluate the molecular mechanism of drugs (13). Network pharmacology can systematically reveal the complex overall biological network relationship among drugs, ingredients, targets and diseases, as well as provide a new perspective for analyzing and predicting the pharmacological mechanism of drugs (14). It has been extensively applied in the mechanistic study of TCM for the treatment of complex diseases (15, 16). Molecular docking is another new method ​used in TCM research, which is one of the most commonly used virtual screening methods and aims to predict the binding conformations of small molecule ligands to the appropriate target binding site (17). It has a significant theoretical meaning to elucidate the mechanism of drugs. Therefore, associating the emerging network science with the characteristics of TCM can promote the transformation of the research strategy of compound TCM from single target to multi-target, combined with experimental verification, to provide a new approach to TCM research.

**Figure 1:**
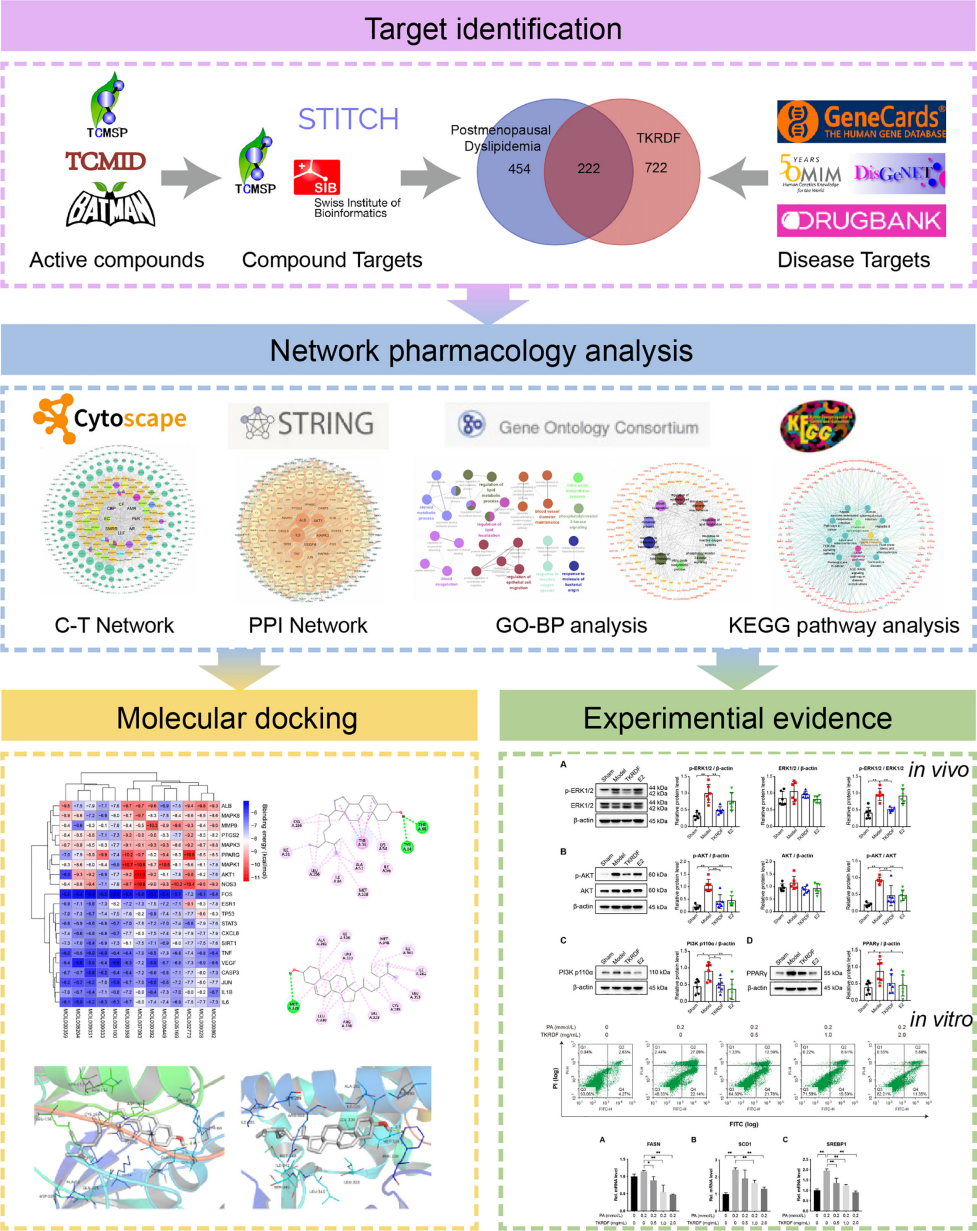
Flowchart of the study.

In this study, an integrated pharmacology strategy employing network pharmacological analysis, molecular docking analysis, and experimental validation was conducted to illustrate the therapeutic mechanism of TKRDF in treating postmenopausal dyslipidemia (**Figure 1**). Briefly, the main objectives of the present study were 1) to screen the active compounds of TKRDF, to predict the potential targets of these compounds against postmenopausal dyslipidemia and to analyze the potential mechanism of the compounds against postmenopausal dyslipidemia by using network pharmacology; 2) to study the binding ability between active compounds of TKRDF and key target genes by using molecular docking; 3) to employ ovariectomy (OVX) and a high-fat diet (HFD) to build an animal model and PA treated cultured hepatocytes to model lipotoxicity to investigate the treatment effect of TKRDF and provide experimental evidence for the potential mechanism. Our study can help in understanding the mechanisms of TKRDF against postmenopausal dyslipidemia and suggest a new approach for investigating the mechanism of action of TCM formulas.

## 2 Materials and Methods

### 2.1 Reagents and Materials

The Chinese traditional medicines, Duzhong (Lot 190602781), Nvzhenzi (Lot 190601), Baizhu (Lot 1909001), Chenpi (Lot 190704821), Zexie (Lot 190702731), Gegen (Lot 190501), Danshen (Lot 1907001), and Shanzha (Lot 190802571), were purchased from Guangdong Kangmei Pharmaceutical Co., Ltd. (Puning, Guangdong, China). The assay kits of total cholesterol (TC), triglyceride (TG), high-density lipoprotein cholesterol (HDL-C), low-density lipoprotein cholesterol (LDL-C), estradiol (E2), follicle stimulating hormone (FSH), interleukin-6 (IL6), tumor necrosis factor-α (TNF-α) and vascular endothelial growth factor A (VEGFA) were purchased from Nanjing Jiancheng Bioengineering Institute (Nanjing, Jiangsu, China). 3-(4,5-Dimethyl-2-thiazolyl)-2,5-diphenyl-2H-tetrazolium bromide (MTT) was purchased from MP Biomedicals, LLC (Solon, OH, USA). Annexin V-FITC apoptosis detection kit was purchased from KeyGen Biotech Co. Ltd. (Nanjing, Jiangsu, China). DMEM/F12 medium, fetal bovine serum (FBS), insulin-transferrin-selenium (ITS) solution, phospho-p44/42 MAPK (ERK 1/2) (Thr202/Tyr204) antibody, p44/42 MAPK (ERK 1/2) antibody, phospho-AKT (Ser473) antibody, AKT antibody, PI3 kinase p110α antibody, and β-actin antibody were purchased from Cell Signaling Technology Inc. (Danvers, MA, USA). PPARγ antibody was purchased from Santa Cruz Biotechnology, Inc. (Dallas, TX, USA). Anti-rabbit and anti-mouse IgG, HRP-linked secondary antibodies were purchased from Cell Signaling Technology Inc. (Danvers, MA, USA). Sodium carboxymethylcellulose (CMC–Na), palmitic acid (PA) and dexamethasone were purchased from Sigma Chemical Co. (St. Louis, MO, USA), 740 Y-P and tBHQ were purchased from MCE (Monmouth, NJ, USA).

### 2.2 Network Pharmacology

#### 2.2.1 Screening Active Compounds of TKRDF

Active compounds of TKRDF were screened using the TCMSP database (Version 2.3, http://lsp.nwu.edu.cn/tcmsp.php), TCMID database (Version 2.0, http://www.megabionet.org/tcmid/) and BATMAN-TCM database (Last update: Jan. 2016, http://bionet.ncpsb.org.cn/batman-tcm/) (18, 19). ADME-related models: Oral bioavailability (OB) ≥ 30%, blood-brain barrier (BBB) ≥ −0.30 and drug-likeness (DL) ≥ 0.18 parameters in TCMSP were used to screen the TCMID and BATMAN-TCM results (20, 21). The final chemical ingredients in TKRDF were the sum of the database results after deduplication.

#### 2.2.2 Screening Compound-Related Targets

Targets of the active compounds were predicted using the TCMSP database, STITCH database (Version 5.0, http://stitch.embl.de/) and Swiss Target Prediction database (http://www.swisstargetprediction.ch/). The 2D chemical structures required by the Swiss Target Prediction database were downloaded from the PubChem database (Last update: Mar. 2019, https://pubchem.ncbi.nlm.nih.gov/). The final compound-related targets of TKRDF were the sum of the database results after deduplication.

#### 2.2.3 Screening Disease-Associated Targets

GeneCards (Version 5.10, https://www.genecards.org/), DisGeNET (Version 7.0, https://www.disgenet.org/), OMIM database (https://omim.org/), and Drugbank database (Version 5.1.9, https://go.drugbank.com/) were used for the identification of potential targets implicated in postmenopausal dyslipidemia. Menopause, and dyslipidemias were used as keywords in the four databases and Homo sapiens targets were selected for research. The intersection of the targets searched by the two keywords was regarded as postmenopausal dyslipidemia-associated targets. The final disease-associated targets were the sum of the database results after deduplication.

#### 2.2.4 Construction of the Compound-Target Network

The Venny 2.1.0 (http://bioinfogp.cnb.csic.es/tools/venny/index.html) online tool was used to draw a Venn diagram of the TKRDF active ingredient target and the postmenopausal dyslipidemia target. The intersection of ingredient-related targets and disease-associated targets was chosen to construct a compound-target network by Cytoscape software (22). The CytoHubba app in Cytoscape software was used to calculate the degree of nodes (23).

#### 2.2.5 Protein–Protein Interaction Analysis

The STRING database (Version 11.5, https://string-db.org/) was chosen to obtain the interactions of proteins. Cytoscape software was used for the development of the PPI network. Cytoscape’s CytoHubba application was used to calculate the degree of protein interaction. The top twenty proteins degrees were selected for further analysis (actually, twenty-one proteins were chosen because the last two proteins had the same degree).

#### 2.2.6 Gene Ontology Enrichment Analysis

Gene ontology (GO) enrichment analysis was performed by the ClueGO application in Cytoscape software (24). During GO-biological process analysis, enrichment/depletion (two-sided hypergeometric test) was used for statistical tests, the *p* value cutoff was 0.05, the kappa score was 0.4, Bonferroni step down was used for the correction method, the Min GO level was 4 and the max GO level was 6, and the cluster rule was the number of genes over 20 and the min percentage over 10.0%.

#### 2.2.7 Kyoto Encyclopedia of Genes and Genomes Pathway Analysis

Kyoto Encyclopedia of Genes and Genomes (KEGG) pathway analysis was performed with the ClueGO application in Cytoscape software. During KEGG pathway analysis, enrichment/depletion (two-sided hypergeometric test) was used for statistical tests, the *p* value cutoff was 0.05, the kappa score was 0.4, Bonferroni step down was used for the correction method, and the cluster rule was number of genes over 30 and the min percentage over 4.0%.

### 2.3 Molecular Docking Analysis

The compound with the highest degree of each Chinese medicine and four mutual compounds in TKRDF were selected as the ligands in the molecular docking, and the top twenty-one major targets from the PPI network analysis were selected as the proteins in the molecular docking. The 3D protein structures were downloaded from the PDB database (https://www.rcsb.org). The water molecules and excess ligands were removed by PyMOL software. The proteins were hydrogenated by AutoDockTools software and exported in pdbqt format. The 3D chemical structures of the ligands were downloaded from the PubChem database, and AutoDockTools software was used to export the files in pdbqt format. After obtaining protein active pockets according to published methods, the molecular docking simulation of potential targets and their corresponding components was carried out by using AutoDock Vina software (25). The binding energy and optimal binding conformation were obtained, R studio was used to draw the heatmap of binding energy, and the docking results were visualized by PyMOL software and Discovery Studio software.

### 2.4 *In Vivo* and *In Vitro* Experiments

#### 2.4.1 Preparation of Tonifying Kidney and Removing Dampness Formula Extract

The crude extract of TKRDF was prepared by the water-extraction and alcohol-precipitation method. Nvzhenzi (420 g), Duzhong (420 g), Gegen (560 g), Shanzha (280 g), Danshen (280 g), Chenpi (280 g), Baizhu (280 g) and Zexie (560 g) mixtures were soaked in 18.48 L of distilled water for 0.5 h and then refluxed for 1 h. The dregs of the herbs were refluxed again in 18.48 L of water for 1 h. Both supernatants were mixed together and filtered using gauze. The supernatant was concentrated to 3.08 L using a rotary evaporator and then 5.28 L of 95% ethanol was added to the extract to make the concentration of ethanol in the extract was 60%. After being placed at 4°C for 24 h, the extract was filtered and concentrated to 1 L using a rotary evaporator. Afterward, the extract was frozen at −80°C for 12 h. The frozen sample was lyophilized using a freeze dryer resulting in 530 g of lyophilized powder.

#### 2.4.2 Animals and Diet

Sprague–Dawley rats (female, 7 weeks old, weighing 250–270 g) were purchased from Guangdong Medical Laboratory Animal Center (Guangzhou, China, Approval No. SCXK (YUE) 2018-0002) and kept in an environmentally controlled room (temperature 22 ± 2°C, humidity 50 ± 10%) with food and water freely available. After one week of adaptation, the rats were subjected to a sham operation or ovariectomized. After 1 week, the rats were fed a normal diet (ND) or a high fat diet (HFD) and assigned to the following four groups: sham-operated + ND (sham group); ovariectomized + HFD (model group); ovariectomized + HFD with TKRDF extract (3.66 g/kg) (TKRDF group); and ovariectomized + HFD with estradiol (0.097 mg/kg) (E2 group). The rats in each group were maintained on these experimental diets for 12 weeks. The composition of the HFD is shown in **Supplementary Table S1**.

TKRDF extract and E2 were dissolved in 0.6% CMC–Na solution and given to the rats by intragastric administration. The treated rats were given the drugs once a day for 12 weeks. The untreated groups (sham group and model group) were given the same volume of 0.6% CMC–Na solution by oral administration once daily for 12 weeks.

#### 2.4.3 Biochemical Serum Analysis

Rat serum was collected from the bloodstream through centrifugation. Serum TC, TG, HDL-C, LDL-C, E2, FSH, IL6, TNF-α, and VEGFA were determined using assay kits, according to the manufacturer’s protocols.

#### 2.4.4 Cell Culture and Treatments

The AML-12 cell line was purchased from National Collection of Authenticated Cell Cultures (Shanghai, China). AML-12 cells were cultured in DMEM/F12 with 10% FBS, 1% ITS solution, and 40 ng/mL dexamethasone, and were incubated in an atmosphere of 95% air and 5% CO_2_ at 37°C. AML-12 cells were used to establish the cellular lipotoxicity model *via* treated with PA.

#### 2.4.5 Cell Viability Assay

AML12 cells were seeded at a density of 5 × 10^3^ cells/well in 96-well plates. After culturing the cells for 24 h, 0.5 mg/mL, 1 mg/mL, or 2 mg/mL TKRDF (dissolved in PBS) was added, and the cells were then incubated for an additional 24 h. PBS was applied to the control group. At the end of TKRDF treatment, 20 μL of MTT (5 mg/mL) was added to each well, and the cells were incubated for 4 h at 37°C. The optical density (OD) at 490 nm was determined using a microplate reader (Eon, BioTek, USA). The experiment was repeated more than three times.

#### 2.4.6 Apoptosis Assay

For the apoptosis experiments, the cells were seeded at a density of 3.0 × 10^5^ cells/well in 6-well plates. AML12 cells were treated with 0.20 mmol/L PA and different concentrations of TKRDF for 24 h. Cell apoptosis analysis was performed using the Annexin V-FITC Apoptosis Detection Kit (KGA107, purchased from KeyGen Biotech Co., Ltd.) or Hoechst 33258 Apoptosis Detection Kit (KGA211, purchased from KeyGen Biotech Co., Ltd.) according to the manufacturer’s protocols. The experiment was repeated three times.

#### 2.4.7 Real-time PCR

Total RNA of the hepatocytes was extracted using TRIzol reagent (Thermo Fisher Scientific). The RNA was reverse-transcribed to cDNA using a HiScriptIIQ RT SuperMix kit (Vazyme, Nanjing, China). Quantitative real-time PCR (qRT–PCR) was performed using ChamQ Universal SYBR qPCR Master Mix (Vazyme, Nanjing, China) according to the manufacturer’s protocol. Relative gene expression was calculated using the 2^−ΔΔCt^ method, and β-actin was used as an internal reference. The primer sequences of β-actin, sterol-regulatory element-binding protein-1c (SREBP-1c), fatty acid synthase (FASN), and stearoyl-coenzyme A desaturase 1 (SCD1) are shown in **Supplementary Table S2**.

#### 2.4.8 Western Blot Analysis

Total protein from either hepatocytes or liver tissues was extracted using RIPA buffer (Cell Signaling Technology Inc., Danvers, MA, USA), and the protein contents was determined using a BCA protein assay kit (Beyotime Biotech Inc., Shanghai, China). After the proteins were quantified, western blot analysis was performed, as described in the literature (26). Densitometry values for the western blot analysis were analyzed by ImageJ Software 1.6 (National Institutes of Health, Bethesda, MD, USA).

#### 2.4.9 Statistical Analysis

Data were analyzed by one-way analysis of variance (ANOVA) followed by Dunnett’s test or a Kruskal–Wallis ANOVA on ranks followed by a Dunn’s test for multiple comparisons and expressed as the means ± standard deviation. All data were analyzed statistically using GraphPad Prism 7.0 (GraphPad Software, Inc., La Jolla, CA, USA), and *p < 0.05 was considered a significant difference.

## 3 Results

### 3.1 Construction of the Compound-Target Network

**Figure 2:**
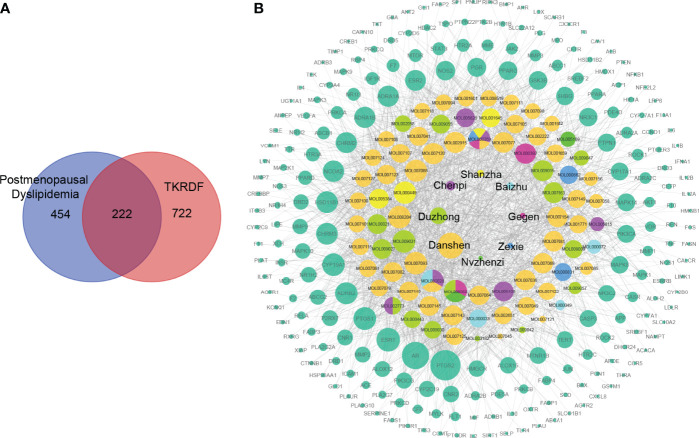
The compound-target network for TKRDF against postmenopausal dyslipidemia. **(A)** Venn diagrams of disease targets and drug targets, two hundred twenty-two overlapping gene symbols between the disease and drug; **(B)** The compound-target network consists of 8 kinds of TCM, 78 compounds, 222 targets including 308 nodes and 1678 edges. The node size is proportional to its degree, and the edge represents the interaction between them.

All of the compounds in TKRDF were obtained from the TCMSP, TCMID, and BATMAN-TCM databases, and three ADME-related models (i.e., OB, BBB and DL) were utilized to filter the active compounds. A total of 78 ingredients (**Table 1**) were recognized in TKRDF. A total of 994 promising targets of the ingredients in TKRDF were identified from the TCMSP, STITCH, and Swiss Target Prediction databases after deleting duplicate targets (**Supplementary Table S3**). A total of 676 postmenopausal dyslipidemia-related targets were collected from the GeneCards, OMIM, Drugbank and DisGeNET databases (**Supplementary Table S4**). As displayed in **Figure 2A**, among the 676 targets related to the disease and the 994 targets related to TKRDF, 222 intersected targets were considered as potential targets for TKRDF to treat postmenopausal dyslipidemia. The compound-target network was built using the Cytoscape software (**Figure 2B**). The network contained 308 nodes and 1678 edges, among which 8 nodes are the Chinses medicines of TKRDF, 78 nodes are the active compounds, and 222 nodes are the target proteins. The CytoHubba app in Cytoscape software was used to calculate the topological features (degree). The size of the node is proportional to the degree. Higher degrees indicated that the compounds or targets played more important roles in TKRDF’s treatment of postmenopausal dyslipidemia.

**Table 1:** Ingredients of TKRDF.

Mol ID	Molecule Name	Chemical Formula	Molecular Weight	OB (%)	BBB	DL	TCM
MOL000028	α-Amyrin	C_30_H_50_O	426.8	39.51	1.28	0.76	Baizhu, Chenpi, Danshen
MOL000359	sitosterol	C_29_H_50_O	414.79	36.91	0.87	0.75	Zexie, Shanzha, Chenpi, Gegen, Danshen
MOL000358	β-sitosterol	C_29_H_50_O	414.79	36.91	0.99	0.75	Duzhong, Nvzhenzi, Gegen
MOL002773	β-carotene	C_40_H_56_	536.96	37.18	1.52	0.58	Chenpi, Duzhong
MOL000033	(3S,8S,9S,10R,13R,14S,17R)-10,13-dimethyl-17-[(2R,5S)-5-propan-2-yloctan-2-yl]-2,3,4,7,8,9,11,12,14,15,16,17-dodecahydro-1H-cyclopenta[a]phenanthren-3-ol	C_30_H_52_O	428.82	36.23	1.09	0.78	Baizhu
MOL000049	3β-acetoxyatractylone	C_17_H_22_O_3_	274.39	54.07	1.08	0.22	Baizhu
MOL000072	8β-ethoxy atractylenolide III	C_17_H_24_O_3_	276.41	35.95	1.12	0.21	Baizhu
MOL000831	Alisol B monoacetate	C_32_H_50_O_5_	514.82	35.58	-0.18	0.81	Zexie
MOL000862	[(1S,3R)-1-[(2R)-3,3-dimethyloxiran-2-yl]-3-[(5R,8S,9S,10S,11S,14R)-11-hydroxy-4,4,8,10,14-pentamethyl-3-oxo-1,2,5,6,7,9,11,12,15,16-decahydrocyclopenta[a]phenanthren-17-yl]butyl] acetate	C_32_H_50_O_5_	514.82	35.58	-0.27	0.81	Zexie
MOL000449	Stigmasterol	C_29_H_48_O	412.77	43.83	1	0.76	Shanzha
MOL001645	Linoleyl acetate	C_20_H_36_O_2_	308.56	42.1	1.08	0.2	Shanzha
MOL005384	suchilactone	C_21_H_20_O_6_	368.41	57.52	0.28	0.56	Shanzha
MOL005100	5,7-dihydroxy-2-(3-hydroxy-4-methoxyphenyl)chroman-4-one	C_16_H_14_O_6_	302.3	47.74	-0.3	0.27	Chenpi
MOL005815	Citromitin	C_21_H_24_O_8_	404.45	86.9	0.16	0.51	Chenpi
MOL005828	nobiletin	C_21_H_22_O_8_	402.43	61.67	-0.08	0.52	Chenpi
MOL000211	Betulinic acid	C_30_H_48_O_3_	456.78	55.38	0.22	0.78	Duzhong
MOL000443	Erythraline	C_18_H_19_NO_3_	297.38	49.18	0.55	0.55	Duzhong
MOL002058	Medioresil	C_21_H_24_O_7_	388.45	57.2	-0.29	0.62	Duzhong
MOL003182	(+)-Medioresinol di-O-beta-D-glucopyranoside_qt	C_21_H_24_O_7_	388.45	60.69	-0.29	0.62	Duzhong
MOL007563	Yangambin	C_24_H_30_O_8_	446.54	57.53	0.01	0.81	Duzhong
MOL009009	(+)-medioresinol	C_21_H_24_O_7_	388.45	87.19	-0.29	0.62	Duzhong
MOL009015	(-)-Tabernemontanine	C_21_H_26_N_2_O_3_	354.49	58.67	0.36	0.61	Duzhong
MOL009027	Cyclopamine	C_27_H_41_NO_2_	411.69	55.42	0	0.82	Duzhong
MOL009030	Dehydrodieugenol	C_20_H_22_O_4_	326.42	30.1	0.63	0.24	Duzhong
MOL009031	Cinchonan-9-al, 6’-methoxy-, (9R)-	C_20_H_24_N_2_O_2_	324.46	68.22	0.17	0.4	Duzhong
MOL009042	Helenalin	C_15_H_18_O_4_	262.33	77.01	-0.22	0.19	Duzhong
MOL009047	(+)-Eudesmin	C_22_H_26_O_6_	386.48	33.29	0.19	0.62	Duzhong
MOL009055	hirsutin_qt	C_18_H_12_O_7_	345.35	49.81	0.02	0.37	Duzhong
MOL009057	liriodendrin_qt	C_22_H_26_O_10_	450.48	53.14	-0.3	0.8	Duzhong
MOL005169	(20S)-24-ene-3β,20-diol-3-acetate	C_32_H_54_O_3_	486.86	40.23	0.58	0.82	Nvzhenzi
MOL000392	formononetin	C_16_H_12_O_4_	268.28	69.67	0.02	0.21	Gegen
MOL001601	1,2,5,6-tetrahydrotanshinone	C_18_H_16_O_3_	280.34	38.75	0.39	0.36	Danshen
MOL001659	Poriferasterol	C_29_H_48_O	412.77	43.83	1.03	0.76	Danshen
MOL001771	poriferast-5-en-3beta-ol	C_29_H_50_O	414.79	36.91	1.14	0.75	Danshen
MOL001942	isoimperatorin	C_16_H_14_O_4_	270.3	45.46	0.66	0.23	Danshen
MOL002222	sugiol	C_20_H_28_O_2_	300.48	36.11	0.7	0.28	Danshen
MOL002651	Dehydrotanshinone II A	C_19_H_16_O_3_	292.35	43.76	0.52	0.4	Danshen
MOL002915	Salvigenin	C_18_H_16_O_6_	328.34	49.07	-0.03	0.33	Danshen
MOL007036	5,6-dihydroxy-7-isopropyl-1,1-dimethyl-2,3-dihydrophenanthren-4-one	C_19_H_22_O_3_	298.41	33.77	0.8	0.29	Danshen
MOL007041	2-isopropyl-8-methylphenanthrene-3,4-dione	C_18_H_16_O_2_	264.34	40.86	0.81	0.23	Danshen
MOL007045	3α-hydroxytanshinoneIIa	C_19_H_18_O_4_	310.37	44.93	0.22	0.44	Danshen
MOL007049	4-methylenemiltirone	C_18_H_18_O_2_	266.36	34.35	0.87	0.23	Danshen
MOL007058	formyltanshinone	C_18_H_10_O_4_	290.28	73.44	-0.28	0.42	Danshen
MOL007061	Methylenetanshinquinone	C_18_H_14_O_3_	278.32	37.07	0.46	0.36	Danshen
MOL007064	przewalskin b	C_20_H_26_O_4_	330.46	110.32	0.22	0.44	Danshen
MOL007069	przewaquinone c	C_18_H_16_O_4_	296.34	55.74	-0.3	0.4	Danshen
MOL007077	sclareol	C_20_H_36_O_2_	308.56	43.67	0.51	0.21	Danshen
MOL007079	tanshinaldehyde	C_19_H_16_O_4_	308.35	52.47	-0.07	0.45	Danshen
MOL007081	Danshenol B	C_22_H_26_O_4_	354.48	57.95	0.11	0.56	Danshen
MOL007082	Danshenol A	C_21_H_20_O_4_	336.41	56.97	-0.01	0.52	Danshen
MOL007085	Salvilenone	C_19_H_22_O_3_	292.4	30.38	1.07	0.38	Danshen
MOL007088	cryptotanshinone	C_19_H_20_O_3_	296.39	52.34	0.51	0.4	Danshen
MOL007093	danshexinkum d	C_21_H_20_O_4_	336.41	38.88	-0.15	0.55	Danshen
MOL007094	danshenspiroketallactone	C_18_H_18_O_3_	282.36	50.43	0.51	0.31	Danshen
MOL007098	deoxyneocryptotanshinone	C_19_H_22_O_3_	298.41	49.4	0.24	0.29	Danshen
MOL007100	dihydrotanshinlactone	C_17_H_14_O_3_	266.31	38.68	0.81	0.32	Danshen
MOL007101	dihydrotanshinoneI	C_18_H_14_O_3_	278.32	45.04	0.43	0.36	Danshen
MOL007105	epidanshenspiroketallactone	C_18_H_20_O_3_	284.38	68.27	0.61	0.31	Danshen
MOL007107	C09092	C_20_H_30_O	286.5	36.07	1.54	0.25	Danshen
MOL007108	isocryptotanshi-none	C_19_H_20_O_3_	296.39	54.98	0.34	0.39	Danshen
MOL007111	Isotanshinone II	C_19_H_18_O_3_	294.37	49.92	0.45	0.4	Danshen
MOL007115	manool	C_20_H_34_O	304.57	45.04	1.16	0.2	Danshen
MOL007118	microstegiol	C_20_H_26_O_2_	298.46	39.61	0.99	0.28	Danshen
MOL007119	miltionone I	C_19_H_20_O_4_	312.39	49.68	-0.11	0.32	Danshen
MOL007120	miltionone II	C_19_H_20_O_4_	312.39	71.03	0.03	0.44	Danshen
MOL007121	miltipolone	C_19_H_24_O	300.43	36.56	0.17	0.37	Danshen
MOL007122	Miltirone	C_19_H_22_O_2_	282.41	38.76	0.87	0.25	Danshen
MOL007123	miltirone II	C_16_H_16_O_4_	272.32	44.95	-0.25	0.24	Danshen
MOL007124	neocryptotanshinone II	C_17_H_18_O_3_	270.35	39.46	0.16	0.23	Danshen
MOL007125	neocryptotanshinone	C_19_H_22_O_4_	314.41	52.49	-0.13	0.32	Danshen
MOL007127	1-methyl-8,9-dihydro-7H-naphtho[5,6-g]benzofuran-6,10,11-trione	C_17_H_12_O_4_	280.29	34.72	-0.27	0.37	Danshen
MOL007143	salvilenone I	C_18_H_22_O_2_	270.4	32.43	0.77	0.23	Danshen
MOL007145	salviolone	C_18_H_20_O_2_	268.38	31.72	0.72	0.24	Danshen
MOL007149	NSC 122421	C_20_H_28_O_2_	300.48	34.49	0.63	0.28	Danshen
MOL007154	tanshinone IIA	C_19_H_18_O_3_	294.37	49.89	0.7	0.4	Danshen
MOL007156	tanshinone VI	C_18_H_16_O_4_	296.34	45.64	-0.28	0.3	Danshen
MOL008204	5-Hydroxy-2’,3’,7,8-tetramethoxyflavone	C_19_H_18_O_7_	358.37	103.11	0.09	0.4	Danshen
MOL008519	neotigogenin	C_27_H_44_O_3_	416.71	80.98	0.23	0.81	Danshen

### 3.2 Construction of the Protein–Protein Interaction Network and Key Targets

**Figure 3:**
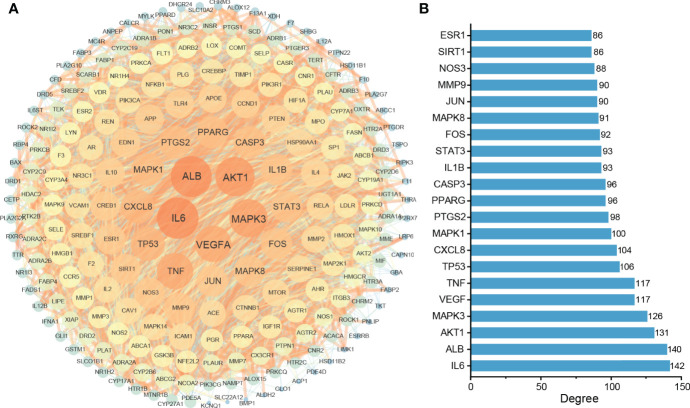
Protein–protein interaction (PPI) network analysis of the target proteins. **(A)** The PPI network consists of 222 nodes and 4364 edges. The size and color of the nodes and edges are proportional to their degree; the higher the degree is, the warmer the color, and the larger the size; **(B)** Top twenty proteins in degrees.

To study the central targets of TKRDF in the treatment of postmenopausal dyslipidemia, the STRING database was used to acquire protein–protein interaction (PPI) relationships among 222 targets, and Cytoscape software was used to further build the PPI network (**Figure 3A**). The network included a total of 222 nodes and 4364 edges, wherein nodes represent the target proteins, while the edges represent the interaction relationships among the proteins. The size and color of the nodes and edges are consistent with their degree and combined score. Among these, the top twenty proteins in the degree are shown in **Figure 3B**, and these were recognized as the core targets of TKRDF in treating postmenopausal dyslipidemia.

### 3.3 Gene Ontology Enrichment Analysis

**Figure 4:**
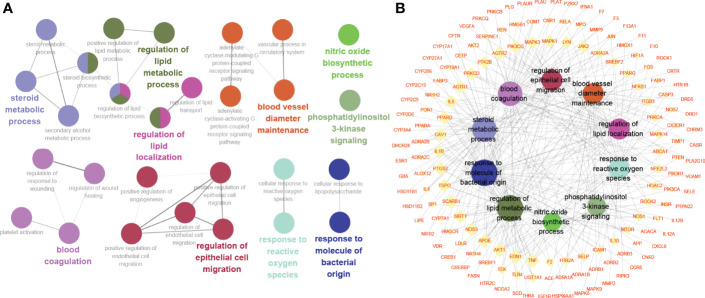
Gene Ontology (GO) enrichment analysis of the target proteins. **(A)** Biosynthetic process analyzed by GO enrichment; **(B)** The target-biosynthetic process network for TKRDF against postmenopausal dyslipidemia.

Gene Ontology enrichment analysis was utilized to clarify the biological functions of the 222 highlighted targets and biosynthetic processes analyzed by the ClueGO app in Cytoscape software. As presented in **Figure 4A**, the biosynthetic processes were clustered into 10 groups after screening, with a total of 28 biosynthetic processes. The most significant biosynthetic processes of each group are highlighted, which included steroid metabolic process, regulation of lipid metabolic process, regulation of lipid localization, phosphatidylinositol 3-kinase signaling, nitric oxide biosynthetic process, response to reactive oxygen species, response to molecule of bacterial origin, blood vessel diameter maintenance, blood coagulation and regulation of epithelial cell migration. The genes involved in these biological processes of TKRDF treatment for postmenopausal dyslipidemia were further analyzed (**Figure 4B**). The top three genes of the ten biosynthetic processes were AKT1, TNF, and EDN1.

### 3.4 Kyoto Encyclopedia of Genes and Genomes Pathway Analysis

**Figure 5:**
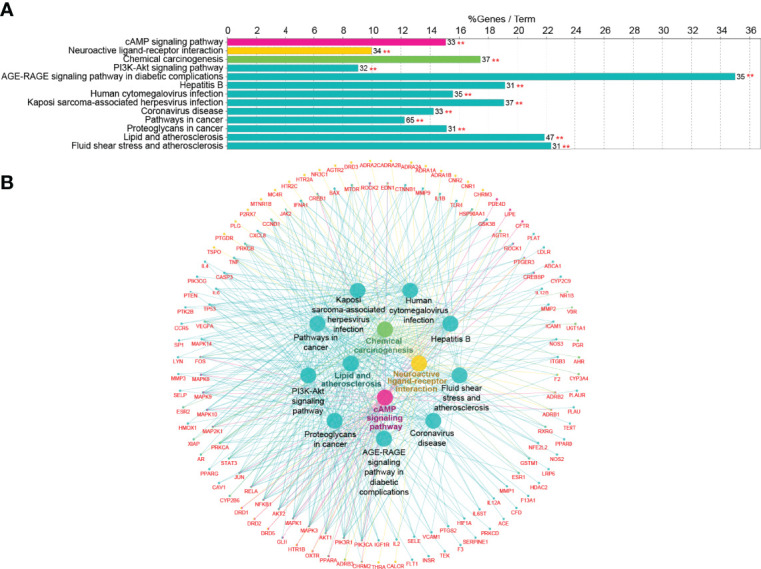
Kyoto Encyclopedia of Genes and Genomes (KEGG) pathway analysis of the target proteins. **(A)** KEGG pathway enrichment; **(B)** The target-pathway network for TKRDF on postmenopausal dyslipidemia. **p < 0.01.

To further identify the potential pathways involved in TKRDF treatment for postmenopausal dyslipidemia, KEGG pathway enrichment analysis of the 222 genes was performed by the ClueGO app in Cytoscape software. As shown in **Figure 5A**, thirteen pathways were obtained after selection, ten pathways were clustered into one group, and the remaining three pathways were clustered into three groups. The target-pathway network is presented in **Figure 5B**. The most significant pathways in each group are highlighted, and the genes involved in these pathways of TKRDF treatment for postmenopausal dyslipidemia were further analyzed. The top five genes of the thirteen pathways were PIK3CA, PIK3R1, AKT1, MAPK3 and MAPK1. Many genes were related to more than two pathways and acted as bridges, which are worthy of our further study.

### 3.5 Molecular Docking Analysis

**Figure 6:**
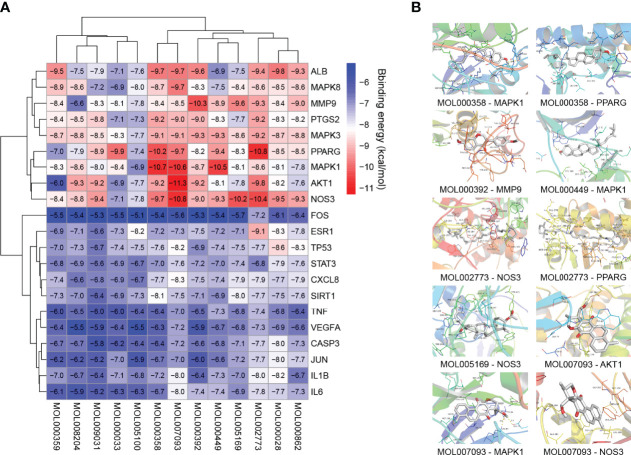
Molecular docking analysis. **(A)** Heatmap of binding energies; **(B)** The detailed protein-compound interactions of the docking simulation.

Twenty-one major targets from the PPI network analysis and thirteen related active compounds from the eight traditional Chinese medicines of TKRDF were selected for molecular docking analysis. Protein structures were obtained from the PBD database through an advanced search, and the TCMSP database was used to obtain the 3D structure from the active compounds. AutoDock Vina software was used to calculate the binding energy between the proteins and the compound ligands. It is believed that the lower the binding energy is, the greater the protein binding affinity of the ligand. As displayed in **Figure 6A**, all of the binding energies were lower than −5.0 kcal/mol, and ten pairs of ingredient-target docking energies were lower than −10.0 kcal/mol, which indicates that their binding conformation seems to be the most stable. The ten protein ligands with the lowest binding energy were selected for further research. PyMOL software was used for 3D visualization, and Discovery Studio software was used to display the receptor–ligand interactions and create a 2D diagram. **Figure 6B** displayed that the binding of most proteins to compounds mainly occurred through hydrogen bond interactions. For example, MOL000358 bound to AKT1 mainly through hydrogen bonding with THR68 and TYR64 and it bound to PPARG mainly through MET923. There were hydrophobic interactions between the proteins and ligands in addition to the hydrogen bond interactions, such as pi hydrophobic interactions, alkyl hydrophobic interactions, and mixed pi/alkyl hydrophobic interactions (**Supplementary Figure S1**). Because the PI3K signaling pathway was mentioned in both GO analysis and KEGG analysis, we conducted molecular docking for 32 genes involved in the PI3K-AKT signaling pathway with the active compounds. The docked compounds revealed good binding affinities with PI3K, AKT and ERK (energy score less than −5 kcal/mol) (**Supplementary Figure S2**). Accordingly, we speculated that the compounds in TKRDF may have a direct impact on the PI3K signaling pathway.

### 3.6 *In Vivo* Experiment

Currently, ovariectomy (OVX) is a recognized model for diseases related to estragon deficiency, and it can simulate the cessation of ovarian function that occurs in postmenopausal women. In this study, we used an ovariectomy and high fat diet (OVX-HFD) model to induce postmenopausal dyslipidemia in female SD rats.

The sham group was the no-treatment sham operation female SD rats, the model group was the no-treatment OVX-HFD female SD rats, the positive drug group (E2 group) was the OVX-HFD female SD rats treated with estradiol (E2) at a dose of 0.097 mg/kg, and the TKRDF group was the OVX-HFD female SD rats treated with TKRDF at a dose of 3.66 g/kg.

The results of the animal experiment are described below.

#### 3.6.1 Effects of TKRDF on Uterus Weight, Serum E2 and Serum FSH Levels

**Figure 7:**
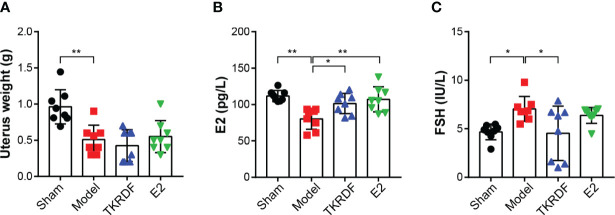
Effects of TKRDF on uterus weight, serum E2 and serum FSH levels. **(A)** Uterus weight of the four groups; **(B)** Serum E2 of the four groups; **(C)** Serum FSH levels of the four groups. Data are means ± SD (n = 8); *p < 0.05; **p < 0.01 vs. Model group.

To confirm that OVX was successfully carried out, the uterus weight, serum E2 and serum FSH concentration were measured (**Figure 7**). Compared with the sham group, the uterine tissue weight was significantly lower in the model, TKRDF and E2 groups because of ovariectomy (*p* < 0.01). Among the groups, the model group had lowest serum E2 and the highest serum FSH, serum E2 levels of the TKRDF and E2 groups were notably increased compared to the model group (*p* < 0.01), and the serum FSH level of the TKRDF group was greatly decreased compared to the model group (*p* < 0.01).

#### 3.6.2 Effects of TKRDF on Body Weight, Liver Weight and Serum Lipid Level

**Figure 8:**
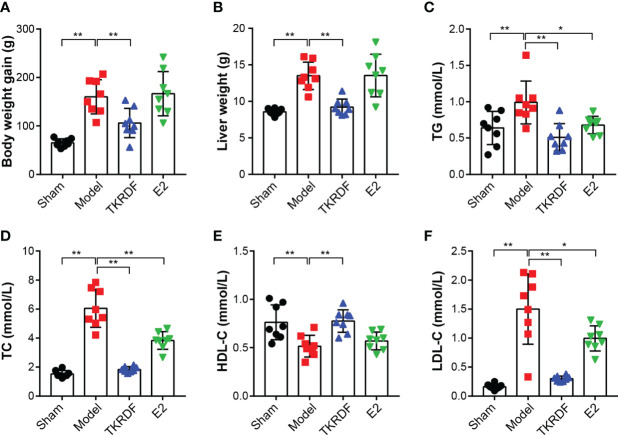
Effects of TKRDF on body weight, liver weight and serum lipid level. **(A)** Body weight gain; **(B)** Liver weight; **(C)** Serum TC level; **(D)** Serum TG level; **(E)** Serum HDL-C level; **(F)** Serum LDL-C level. Data are means ± SD (n = 8); *p < 0.05; **p < 0.01 vs. Model group.

The body weight of the rats was measured, and weight gain was calculated. The weight of the liver was weighed at the end of the experiment, and serum TC, TG, HDL-C and LDL-C levels were measured. The results are displayed in **Figure 8**. After ovariectomy and high-fat diet induction, body weight gain was significantly higher in the model group than in the sham group (*p* < 0.01). Compared to the model group, the weight gain of the TKRDF group was significantly decreased (*p* < 0.01). Meanwhile, a significant difference in liver weight was observed among the groups (F (3, 28) = 9.093, *p* = 0.0002), the liver weight of the model group was significantly increased compared to that of the sham group (*p* < 0.01), and the liver weight of the TKRDF group was significantly reduced compared to that of the model group. Moreover, ovariectomy and a high-fat diet markedly increased the TC, TG and LDL-C levels of the model group and markedly decreased their HDL-C levels. TKRDF treatment demonstrated a positive effect on serum lipid levels, it not only decreased TCHO, TG and LDL-C levels but also increased HDL-C levels. Compared with the model group, the E2 group had lower TC and TG levels and higher HDL-C levels (*p* < 0.01). Although the LDL content of the E2 group was lower than that of the model group, the difference was not statistically significant. In summary, these data indicated that TKRDF was able to protect against postmenopausal dyslipidemia in rats.

#### 3.6.3 Effects of TKRDF on Serum IL6, TNF-α and VEGFA Levels

**Figure 9:**
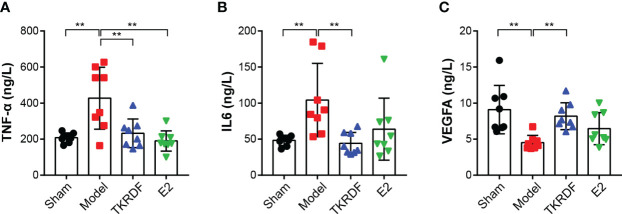
Effects of TKRDF on serum IL6, TNF-a and VEGFA levels. **(A)** Serum IL6 level; **(B)** Serum TNF-a level; **(C)** Serum VEGFA level. Data are means ± SD (n = 8); **p < 0.01 vs. Model group.

IL6, TNF-α and VEGFA cytokines were major proteins in the PPI network; therefore, IL6, TNF-α and VEGFA contents in the serum were tested. As presented in **Figure 9**, IL6 and TNF-α significantly increased (*p* < 0.01) after ovariectomy and a high-fat diet for 12 weeks, and VEGFA was distinctly lowered (*p* < 0.01). TKRDF treatment reduced the content of IL6 and TNF-α in the serum and increased the content of VEGFA in the serum (*p* < 0.01). E2 treatment had a similar effect, but the changes in the levels of IL6 and VEGFA did not reach significance. These results suggested that TKRDF reduces proinflammation cytokines and promotes angiogenesis *in vivo*.

#### 3.6.4 Effect of TKRDF on ERK1/2, AKT, PI3K p110α and PPARγ Protein Expression in Liver Tissue

**Figure 10:**
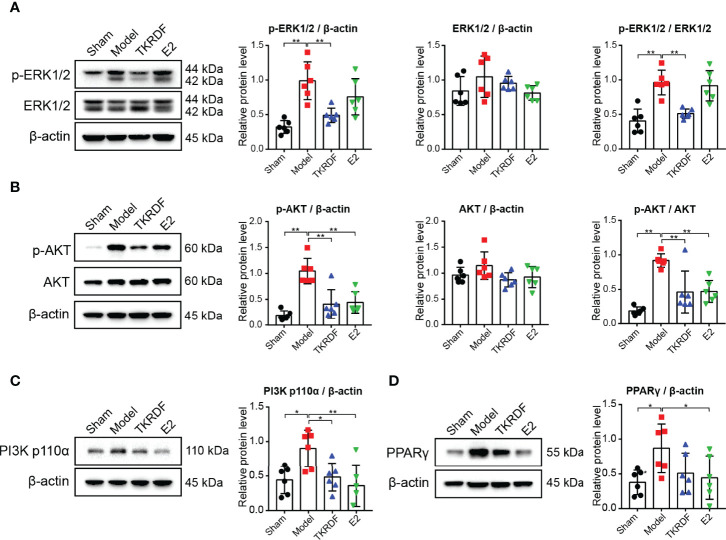
Effect of TKRDF on ERK1/2, AKT, PI3K p110α and PPARγ expression in liver tissue. **(A)** Protein expression levels of p-ERK1/2 and ERK1/2 in the liver tissue; **(B)** Protein expression levels of p-AKT and AKT in the liver tissue; **(C)** Protein expression levels of PI3K p110a in the liver tissue; **(D)** Protein expression levels of PPARγ in the liver tissue. Data are means ± SD (n = 6); *p < 0.05; **p < 0.01 vs. Model group.

To study the regulation of proteins in TKRDF-treated postmenopausal dyslipidemia *in vivo*, western blot analysis was used to determine the expression of ERK1/2 (MAPK3/1), AKT, PI3K p110α (PIK3CA) and PPARγ (PPARG) in liver tissue. **Figure 10A** displayed that there were no notable differences in ERK1/2 expression levels among the four groups. The ERK1/2 phosphorylation level of the model groups tripled compared to that of the sham group (p < 0.001). Compared with the model group, the ERK1/2 phosphorylation of the rats was significantly decreased after 12 weeks of treatment with TKRDF (*p* < 0.001). The variation trend of AKT expression in the liver tissue was similar to that of the ERK1/2 protein (**Figure 10B**). In contrast, the levels of AKT protein in the liver were not different among the four groups; the model group had the highest AKT phosphorylation level, and TKRDF and E2 treatment obviously suppressed AKT phosphorylation levels (*p* < 0.001). Meanwhile, the model group showed significantly increased PI3K p110α and PPARγ (*p* < 0.05) protein expression compared with the sham group (**Figures 10C, D** **)**, whereas TKRDF (*p* < 0.05) and E2 (*p* < 0.001) treatment effectively reversed the change in PI3K p110α expression. Compared with the model group, PPARγ expression was reduced in the treatment groups, but the change in the level of PPARγ in the TKRDF group did not reach significance. In general, the effect of TKRDF in preventing postmenopausal dyslipidemia might be related to the ERK1/2 and PI3K/AKT signaling pathways.

### 3.7 *In Vitro* Experiment

Liver metabolic health is strongly linked to blood lipid levels in postmenopausal women (27). Dyslipidemia and the resulting lipotoxicity often result in lipotoxic injuries to hepatocytes. The AML-12 cell line, an immortalized normal mouse hepatocyte cell line, is often used in lipotoxicity studies of cultured hepatocytes *in vitro* (28). To understand the mechanistic contributions of TKRDF to liver injury induced by postmenopausal dyslipidemia, lipotoxicity was modeled by exposing AML12 cells to PA in this study.

The results of the cellular experiments are described below.

#### 3.7.1 Effect of TKRDF on Cell Viability in PA-Treated AML12 Cells

**Figure 11:**
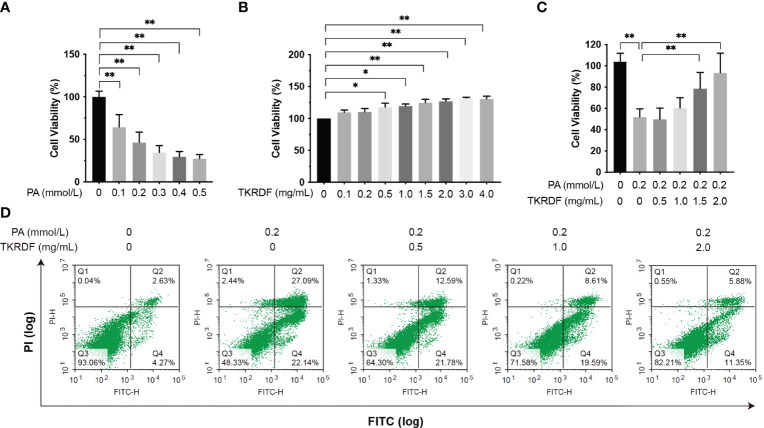
Effect of TKRDF on cell viability in PA-treated AML12 cells. **(A)** Cell viability of PA-treated AML12 cells by MTT assay (n = 15); **(B)** Cell viability of TKRDF-treated AML12 cells by MTT assay (n = 6); **(C)** Cell viability of TKRDF on PA-treated AML12 cells by MTT assay (n = 6); **(D)** Representative flow cytometry profiles (Annexin V-FITC/PI staining) for AML12 cells after various treatments (n = 3). Region Q1: damaged cells (PI-positive/Annexin V-negative); region Q2: late apoptotic and dead cells (PI-positive/Annexin V-positive); region Q3: early apoptotic cells (PI-negative/Annexin V-positive); and region Q4: vital cells (PI-negative/Annexin V-negative). Data are means ± SD; *p < 0.05; **p < 0.01.

First, we investigated the cell viability of AML12 cells treated with various concentrations of PA for 24 h and based on these results, 0.2 mmol/L was selected for subsequent experiments (**Figure 11A**). Meanwhile, the cytotoxicity of various concentrations of TKRDF toward AML12 cells was investigated using MTT assays (**Figure 11B**), and the results showed that TKRDF concentrations below 4.0% were not cytotoxic to AML12 cells. PA-damaged AML12 cells were given TKRDF at concentrations of 0.5, 1.0, 1.5, and 2.0 mg/mL (*p* < 0.05). As shown in **Figure 11C**, in PA-induced murine hepatic AML12 cell injury, 1.5 and 2.0 mg/mL TKRDF alleviated the hepatocellular lipotoxicity caused by PA (*p* < 0.05). Additionally, the effect of TKRDF on apoptosis in PA-treated AML12 cells was investigated using a flow cytometry assay (Annexin V-FITC/PI staining). As shown in **Figure 11D**, PA-treated AML12 cells exhibited 49.23% apoptosis, and 0.5, 1.0 and 2.0 mg/mL TKRDF treatment decreased the percentage of apoptosis to 34.27%, 28.2% and 17.23%, respectively. This result further verifies the protective effect of TKRDF against AML12 cell lipotoxicity caused by PA.

#### 3.7.2 Effect of TKRDF on the mRNA Levels of Lipid Metabolism Genes in PA-Treated AML12 Cells

**Figure 12:**
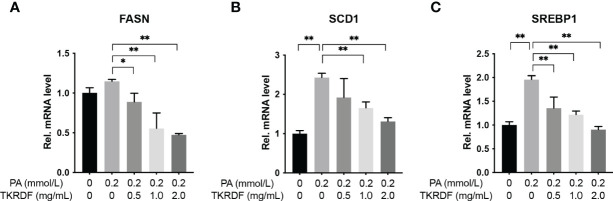
Effect of TKRDF on the mRNA levels of lipid metabolism genes in PA-treated AML12 cells. **(A)** Relative mRNA levels of FASN; **(B)** Relative mRNA levels of SCD1; **(C)** Relative mRNA levels of SREBP1. Data are means ± SD (n = 3); *p < 0.05; **p < 0.01.

To unveil the mechanisms by which TKRDF affects lipotoxicity, we assessed the effect of TKRDF on the expression of lipid metabolism-related genes, which are key enzymes involved in fatty acid synthesis, including FASN; unsaturated fatty acid synthesis-catalyzing enzymes, including SCD1; and transcription factors involved in lipogenesis, including SREBP1. The results are shown in **Figure 12**. The mRNA levels of FASN, SCD1 and SREBP1 were increased in PA-treated AML12 cells compared to those in normal AML12 cells, and the elevations in SCD1 and SREBP1 were statistically significant (*p* < 0.05). TKRDF downregulated the mRNA expression levels of FASN, SCD1 and SREBP1 (*p* < 0.05). These data suggest that TKRDF might inhibit lipotoxicity by reducing the transcription of lipid synthesis genes.

#### 3.7.3 Effect of TKRDF on ERK1/2, AKT and PI3K p110α Protein Expression in PA-Treated AML12 Cells

**Figure 13:**
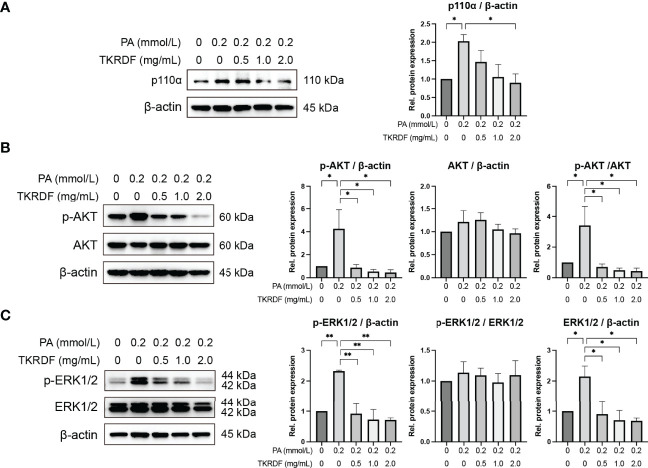
Effect of TKRDF on ERK1/2, AKT and PI3K p110α protein expression in PA-treated AML12 cells. **(A)** Protein expression of PI3K p110a; **(B)** Protein expression of p-AKT and AKT; **(C)** Protein expression of p-ERK1/2 and ERK1/2. Data are means ± SEM (n = 3); *p < 0.05; **p < 0.01.

Based on our network pharmacology, molecular docking and *in vivo* findings, PI3K p110α, AKT and ERK1/2 seem to be very important in TKRDF’s treatment of menopausal dyslipidemia. Therefore, we also determined the protein expression levels of ERK1/2, AKT and PI3K p110αl in PA-treated AML12 cells by western blot. **Figure 13** shown that there were no notable differences in AKT and ERK1/2 expression levels among the five groups of cells. AML12 cell PA stimulation was administered, resulting in an increase in PI3K p110α expression and an increase in the phosphorylation levels of AKT and ERK1/2 (*p* < 0.05). TKRDF dose-dependently inhibited the expression of PI3K p110α, phosphorylated AKT and phosphorylated ERK1/2 in PA-treated AML12 cells (*p* < 0.05). The western blot results further confirmed the regulatory effect of TKRDF on PI3K p110α, AKT and ERK1/2. Besides, PI3K agonist 740 Y-P (20 μmol/L) and ERK agonist t-butylhydroquinone (tBHQ, 50 μmol/L) treatment reversed TKRDF induced cell viability enhanced and apoptosis decreased in PA-treated AML12 cells (**Supplementary Figure S3**). This evidence indicated that TKRDF against AML12 cell lipotoxicity caused by PA through downregulating PI3K/AKT signaling pathway and MAPK cascade.

## 4 Discussion

The lack of estrogen is a major factor in the onset of cardiovascular disease after menopause, and one of its characteristics is changes in blood lipids (29). Management of menopausal dyslipidemia is the main approach to reduce the incidence of cardiovascular disease in postmenopausal women (6). Menopause hormone therapy (MHT) is an effective strategy for women with menopause-related symptoms. However, the data overall do not support the use of MHT for the primary prevention of cardiovascular disease (30–32). A previous study indicated that TKRDF reduced the lipid profile in Chinese postmenopausal women with mild dyslipidemia (10). However, the underlying mechanism of how TKRDF treats postmenopausal dyslipidemia remains unclear. Network pharmacology is a promising method for understanding TCM formulas and predicting and identifying multiple drug targets and interactions in diseases (33). In the present research, a network pharmacology-based strategy combined with molecular docking studies and experiment validation was employed to investigate bioactive compounds, potential targets, and the molecular mechanism of TKRDF against postmenopausal dyslipidemia.

According to the network pharmacology analysis, we obtained 78 active compounds from TKRDF that acted on 222 targets of postmenopausal dyslipidemia. The active compounds were mainly terpenoids, lignans, and flavonoids. Most of these compounds have been found to have positive effects on the cardiovascular system, menopause and dyslipidemia. Among the active compounds, most diterpenoids were found in Danshen, such as tanshinone IIA, which exerts anti-inflammatory and antioxidant effects and affects HDL subfractions to attenuate cardiovascular diseases such as atherosclerosis (34, 35). Triterpenoids, especially pentacyclic triterpenes, are regarded as a new tool to fight metabolic syndrome, and α-Amyrin was found to attenuate high fructose diet-induced high TC, high TG, and metabolic syndrome in rats (36). Betulinic acid also has antihyperlipidemic activities (37). Certain lignans termed phytoestrogens (38), such as stigmasterol, are associated with a favorable metabolic cardiovascular risk profile (39). Flavonoids, such as nobiletin, were reported to scavenge free radicals, improve glucose tolerance and insulin sensitivity, modulate lipid metabolism and adipocyte differentiation, suppress inflammation and apoptosis, and ameliorate endothelial dysfunction (40). Therefore, all of these findings showed that multiple components of TKRDF had a positive effect on postmenopausal dyslipidemia, which was confirmed by the *in vivo* and *in vitro* experimental results. In the *in vivo* verification experiment, the levels of estrogen and follicle stimulating hormone were balanced in the TKRDF treatment, and the body weight, liver weight, and blood lipids were reduced. In PA-induced murine hepatic AML12 cell injury, TKRDF reduced the lipotoxicity of hepatocytes and inhibited the expression of lipid synthesis genes. These data confirmed the effectiveness of TKRDF in the treatment of postmenopausal dyslipidemia.

Obesity and dyslipidemia are associated with low-grade inflammation in the body, and improving the inflammatory status was demonstrated to ameliorate dyslipidemia in postmenopausal women (41). The results of the PPI network analysis revealed that the proinflammatory cytokines IL6 and TNF-α interacted closely with other genes and were at the core of the network. IL6 and TNF-α play an important role in the development of inflammation (42). In addition, IL-6 is one of the adipokines that promotes insulin resistance and dyslipidemia in humans, and TNF-α is the driver behind dyslipidemia (43). VEGFA is another key gene in the PPI network; it regulates proliferation, angiogenesis, and the migration of endothelial cells, increases vascular permeability, and controls thrombogenicity (44). Maintaining appropriate IL6, TNF-α and VEGFA levels is important to maintain normal blood lipids and vascular homeostasis in postmenopausal women. In the *in vivo* verification experiment, TKRDF downregulated IL6 and TNF-α and upregulated VEGFA. Hence, we speculated that TKRDF might have the ability to regulate inflammation and promote angiogenesis and thus maintain normal blood lipids and vascular homeostasis.

The liver plays an important role in lipid synthesis, lipid metabolism, and lipid uptake. Currently, lipid-lowering drugs targeting the liver can treat hyperlipidemia, and cardiovascular benefits have been found (45). The network pharmacology studies showed that FASN, SCD1 (SCD) and SREBP1 (SREBF1) are targets for the treatment of postmenopausal dyslipidemia by TKRDF. In hepatic AML12 cells, the regulatory role of TKRDF in these genes has also been confirmed. SCD1, SREBP1 and FASN play important roles in lipid synthesis. The SCD1 gene encodes stearoyl-CoA desaturase, which converts saturated fatty acids into monounsaturated fatty acids. Mice with SCD1 deficiency had better insulin sensitivity and reduced liver fat (46). The mature form of SREBP1 is a transcription factor involved in lipid synthesis, which participates in Toll-like receptor 4-triggered inflammatory pathways during the resolution phase of inflammation in macrophages. FASN is a key enzyme for endogenous fatty acid synthesis. FASN converts excess carbohydrates into fatty acids, further esterifying them into triacylglycerol. As a downstream effector, FASN can be activated by the PI3K/AKT/mTOR signaling pathway and transcription factors such as SREBP-1, ZBTB7A, and p53. These findings could explain why TKRDF can reduce PA-induced abnormal lipid metabolism and lipotoxicity in hepatocytes.

A variety of protein kinases participate in lipid metabolism, such as mitogen-activated protein kinase (MAPK) and protein kinase B (AKT) (47). ERK1/2 (MAPK3/1) signaling has been previously revealed to regulate SREBP-2 activity *via* direct phosphorylation, thereby regulating hepatic very low-density lipoprotein secretion and the subsequently circulating LDL-C and TG levels (48). In our study, ERK1 (MAPK3) and ERK2 (MAPK1) had abundant interactions with other genes in the PPI network and were associated with various biological processes and signaling pathways in the GO and KEGG analyses. Results from molecular docking showed that the active components of TKRDF had a strong interaction with ERK (MAPK3 and MAPK1). Some compounds in TKRDF have been reported to affect ERK. α-Amyrin and β-carotene played anti-inflammatory effect *via* inhibition of ERK signaling pathway (49, 50). Formononetin decreased apoptosis by inhibiting phosphorylation of ERK (51). Based on the network pharmacology and molecular docking results, we speculated that ERK was the main targets of TKRDF against postmenopausal dyslipidemia. Hence, we measured ERK1/2 expression in rat liver and AML12 cells and found that TKRDF reduced ERK1/2 phosphorylation, and ERK agonist antagonized the positive effects of TKRDF in PA-treated AML12 cells. ERK1/2 has been reported to be involved in many metabolic diseases by regulating inflammation, reducing lipids, and improving insulin sensitivity and glucose tolerance (52). Inhibition of the ERK1/2 signaling pathway would be expected to alleviate postmenopausal dyslipidemia. These findings implied that the positive effects of TKRDF in postmenopausal dyslipidemia resulted from attenuation of the MAPK cascade.

In addition, the GO enrichment analysis showed that the pharmacological effects of TKRDF on postmenopausal dyslipidemia were related to phosphatidylinositol 3-kinase signaling. The target points in the KEGG enrichment analysis were also enriched in the PI3K/AKT signaling pathway. The PI3K/AKT pathway was identified in this work as another signaling pathway worthy of attention. Recent studies have proposed a potential link between the PI3K/AKT pathway and dyslipidemia, and PI3K/AKT activation enhances SREBP-1 activity, further regulating the enzymes involved in the synthesis of cholesterol and TG in the liver and other tissues, which leads to dyslipidemia (53). Pharmacological inhibition of PI3K is an effective and safe anti-obesity intervention that could reverse the negative effects of metabolic syndrome in humans (54). TKRDF was found to reduce PI3K p110α expression and AKT phosphorylation in experimental validation, and PI3K agonist reversed the positive effects of TKRDF. The active compounds that bound to PI3K and AKT had good docking results. Several compounds in TKRDF have been reported to affect the PI3K/AKT signaling pathway. β-sitosterol downregulated the expression of apoptosis-related genes through the PI3K/Akt pathway and attenuates liver injury in a rat model of chronic alcohol intake (55). Consequently, it is speculated that the PI3K/Akt signaling pathway is one of the mechanisms by which TKRDF treats postmenopausal dyslipidemia.

According to previous research, the phosphorylation of ERK1/2 promotes PPARγ overexpression in the liver, which increases TG accumulation and the development of hepatic steatosis (47). The results of network pharmacology and molecular docking indicated that PPARγ seems to be one of the targets of TKRDF in the treatment of postmenopausal dyslipidemia. Experimental verification found that TKRDF indeed reduced the expression of PPARγ in the liver, but there was no significant difference. More experiments might be needed to determine the effect of TKRDF on PPARγ, such as detecting the phosphorylation of PPARγ and verifying the expression of PPARγ in adipose tissue.

In summary, based on the results of network pharmacology, molecular docking, and experimental validation, our study suggested that TKRDF ameliorates postmenopausal dyslipidemia through multiple components, targets, and pathways. However, the present study still has several limitations. First, the active compounds of TKRDF were obtained from a variety of databases, and these results may not accurately reflect the absorption and utilization of TKRDF *in vivo*. More proof is needed to verify the mechanism of TKRDF in treating postmenopausal dyslipidemia.

## 5 Conclusion

In the present study, we combined network pharmacology prediction, molecular docking, and *in vivo* and *in vitro* experiments to research the active ingredients, potential targets, and potential mechanism of TKRDF against postmenopausal dyslipidemia. The results suggest that TKRDF ameliorates postmenopausal dyslipidemia by regulating hormone levels, inhibiting inflammation, promoting angiogenesis and inhibiting lipid synthesis. These effects appear to be related to TKRDF affecting the ERK and PI3K/AKT signaling pathways. This work supplies a foundation for the treatment of endocrine disorder-related complex diseases with TCM and provides support for the clinical application of TKRDF. Meanwhile, this study proposed a feasible method to reveal the scientific basis and therapeutic mechanism of TCM in the treatment of diseases.

## Data Availability Statement

The original contributions presented in the study are included in the article/**Supplementary Material**. Further inquiries can be directed to the corresponding authors.

## Ethics Statement

The animal study was reviewed and approved by Institutional Animal Care and Use Committee of Guangdong Provincial Hospital of Chinese Medicine, Guangzhou University of Chinese Medicine.

## Author Contributions

GN and JuL conceived and designed the study. XL performed network pharmacology research, molecular docking, and cell experiments and drafted the manuscript. HC performed the animal experiments and cell experiments and analyzed the data. HY, JiL and YL helped analyze the data and prepare the manuscript. YD, LW and JW helped conduct the experiment. GN and JuL revised the manuscript and provided funding support. All authors confirmed the final manuscript. XL and HC have contributed equally to this work and share first authorship.

## Funding

This research was funded by the National Natural Science Foundation of China (No. 81804132), Guangdong Basic and Applied Basic Research Foundation (No. 2022A1515011651; No. 2021A1515220139; No. 2021A1515012573), Science and Technology Foundation of Guangzhou City (No. 202102010257), State Key Laboratory of Dampness Syndrome of Chinese Medicine Research Foundation (No. SZ2021ZZ21; No. SZ2021ZZ47), Research Fund for Bajian Talents of Guangdong Provincial Hospital of Chinese Medicine (No. BJ2022KY09), Specific Research Fund for TCM Science and Technology of Guangdong Provincial Hospital of Chinese Medicine (No. YN2019MJ15), and Scientific research projects of Guangdong Bureau of traditional Chinese Medicine (No. 20222086).

## Conflict of Interest

The authors declare that the research was conducted in the absence of any commercial or financial relationships that could be construed as a potential conflict of interest.

## Publisher’s Note

All claims expressed in this article are solely those of the authors and do not necessarily represent those of their affiliated organizations, or those of the publisher, the editors and the reviewers. Any product that may be evaluated in this article, or claim that may be made by its manufacturer, is not guaranteed or endorsed by the publisher.
