# Supplementary material for: Study on the Potential Mechanism of Tonifying Kidney and Removing Dampness Formula in the Treatment of Postmenopausal Dyslipidemia Based on Network Pharmacology, Molecular Docking and Experimental Evidence
Source: Front Endocrinol (Lausanne). 2022 Jul 7;13:918469. doi: 10.3389/fendo.2022.918469 (PMC9302042; doi:10.3389/fendo.2022.918469)
Supplement: Supplementary file 1 [file DataSheet_1.docx]

Supplementary Material

# Supplementary Tables

**Supplementary Table S1:** Compositions of the High Feed Diet.

| **Nutrients** | **Mass percent (%)** | **Energy ratio (%)** |
| --- | --- | --- |
| Protein | 19 | 17 |
| Carbohydrate | 50 | 46 |
| Fat | 18 | 37 |
| TOTAL | 87 | 100 |
| **Energy: 4.4 kcal/g** | | |
| **Materials** | **Mass ratio (g/kg)** | **kcal** |
| Casein | 100 | 400 |
| Sucrose | 200 | 800 |
| Lard | 150 | 1350 |
| Rats and mice maintain compound feed | 522 | 1879 |
| Special premix for rats and mice | 4 | 0 |
| Cholesterol | 12 | 0 |
| Sodium Cholate | 2 | 0 |
| Calcium hydrophosphate (feed grade type I) | 6 | 0 |
| Limestone (calcium carbonate) | 4 | 0 |
| TOTAL | 1000 | 4429 |

**Supplementary Table S2:** Sequence of primers.

| **Gene** | **NCBI Gen Bank** | **Forward Primers (5’→3’)** | **Reverse Primers (5’→3’)** |
| --- | --- | --- | --- |
| FASN | NM_007988.3 | GGAGGTGGTGATAGCCGGTAT | TGGGTAATCCATAGAGCCCAG |
| SCD1 | NM_009127.4 | TTCTTGCGATACACTCTGGTGC | CGGGATTGAATGTTCTTGTCGT |
| SREBP1 | NM_001313979.1 | CTTTGGCCTCGCTTTTCGG | TGGGTCCAATTAGAGCCATCTC |
| β-actin | NM_007393.5 | GTGTGCACTTTTATTGGTCTCAA | GGAGGGGGTTGAGGTGTT |

**Supplementary Table S3:** Compound-Related Targets.

| **Compound-Related Targets** |
| --- |
| ABAT, ABCA1, ABCB1, ABCB11, ABCC1, ABCC9, ABCG2, ABCG5, ABCG8, ABHD6, ABL1, ACACA, ACACB, ACE, ACE2, ACHE, ACKR3, ACP1, ACPP, ACVRL1, ADA, ADAM17, ADAM33, ADAM9, ADAMTS4, ADAMTS5, ADCY10, ADCY5, ADH1C, ADORA1, ADORA2A, ADORA2B, ADORA3, ADRA1A, ADRA1B, ADRA1D, ADRA2A, ADRA2B, ADRA2C, ADRB1, ADRB2, ADRB3, AGPAT2, AGTR1, AGTR2, AHR, AHSA1, AKR1A1, AKR1B1, AKR1B10, AKR1C1, AKR1C2, AKR1C3, AKR1C4, AKR1E2, AKT1, AKT2, AKT3, ALB, ALDH1A1, ALDH2, ALDH3A1, ALK, ALOX12, ALOX15, ALOX5, ALOX5AP, ALPG, ALPL, AMPD1, AMPD2, AMPD3, AMY1A, ANPEP, AOC3, APEX1, APH1A, APH1B, APOE, APP, AR, ARG1, ASIC3, ATAD2, ATF3, ATG12, ATM, ATP12A, ATP2A1, ATP5F1B, AURKA, AURKB, AVPR1A, AVPR2, AXL, BACE1, BACE2, BAX, BAZ2A, BAZ2B, BCHE, BCL2, BCL2L1, BCL2L11, BCMO1, BCO2, BDKRB1, BDKRB2, BIRC2, BIRC3, BIRC5, BLK, BMP1, BMX, BRAF, BRD2, BRD3, BRD4, BRF1, BRPF1, BRS3, C5AR1, CA1, CA12, CA13, CA14, CA2, CA3, CA4, CA5A, CA5B, CA6, CA7, CA9, CACNA1B, CACNA1C, CACNA1G, CACNA2D1, CALCR, CALCRL, CALM1, CALM3, CAMK2D, CAMK2G, CAPN1, CAPN10, CASP1, CASP3, CASP6, CASP7, CASP8, CASP9, CASR, CAV1, CBR1, CCKAR, CCKBR, CCNA1, CCNA2, CCNB1, CCNB2, CCNB3, CCNC, CCND1, CCNE1, CCNE2, CCNT1, CCR1, CCR4, CCR5, CCR9, CD163, CD38, CD81, CDC25A, CDC25B, CDC25C, CDC7, CDK1, CDK2, CDK3, CDK4, CDK5, CDK5R1, CDK6, CDK7, CDK8, CDK9, CDKN1A, CES1, CES2, CETP, CFD, CFLAR, CFTR, CHEK1, CHRM1, CHRM2, CHRM3, CHRM4, CHRM5, CHRNA2, CHRNA3, CHRNA4, CHRNA5, CHRNA6, CHRNA7, CHRNB2, CHRNB3, CHRNB4, CHUK, CLEC2D, CLK1, CLK3, CLK4, CMA1, CNR1, CNR2, COMT, CPA1, CPB1, CREB1, CREBBP, CRHR1, CSF1R, CSK, CSNK1G1, CSNK2A1, CTNNB1, CTRB1, CTRC, CTSA, CTSB, CTSC, CTSG, CTSK, CTSL, CTSS, CTSV, CX3CR1, CXCL8, CXCR1, CXCR2, CYCS, CYP11B1, CYP11B2, CYP17A1, CYP19A1, CYP1A1, CYP1A2, CYP1B1, CYP24A1, CYP27A1, CYP2B6, CYP2C19, CYP2C9, CYP2D6, CYP3A4, CYP51A1, CYP7A1, DAPK1, DAPK2, DAPK3, DDX20, DGAT1, DHCR24, DHCR7, DHFR, DHH, DHODH, DNAH8, DNM1, DNMT1, DNMT3A, DNTT, DPP4, DPP7, DPP8, DPP9, DRD1, DRD2, DRD3, DRD4, DRD5, DUSP1, DUSP3, DUSP6, DUT, DYRK1A, DYRK1B, DYRK2, EBP, ECE1, EDN1, EDNRA, EDNRB, EED, EGFR, EGLN1, EIF2AK3, EIF4H, ELANE, ELAVL1, EP300, EPAS1, EPHA2, EPHA5, EPHB2, EPHB4, EPHX1, EPHX2, ERBB2, ERBB4, ERCC2, ERCC3, ERCC5, ERG, ERN1, ESR1, ESR2, ESRRA, ESRRB, ESRRG, EZH2, F10, F11, F12, F13A1, F2, F2R, F2RL1, F3, F7, FAAH, FABP1, FABP2, FABP3, FABP4, FABP5, FADS1, FAP, FARS2, FASN, FBP1, FCER2, FDFT1, FEN1, FFAR1, FGF1, FGF2, FGFR1, FGFR3, FKBP1A, FLT1, FLT3, FLT4, FN1, FNTA, FNTB, FOLH1, FOS, FUT4, FUT7, FYN, FZD1, G6PD, GABBR1, GABBR2, GABRA1, GABRA2, GABRA3, GABRA5, GABRA6, GABRB2, GABRB3, GABRE, GABRG2, GABRG3, GBA, GBA2, GCGR, GCK, GEMIN2, GHSR, GJA1, GLI1, GLO1, GLP1R, GLRA1, GLRA2, GPBAR1, GPER1, GPR119, GPR139, GPR142, GPR35, GPR39, GPR55, GPR88, GRIA2, GRIN1, GRIN2A, GRIN2B, GRK2, GRK3, GRK5, GRK6, GRM1, GRM2, GRM4, GRM5, GSK3A, GSK3B, GSTA1, GSTM1, GUSB, HAO2, HCAR2, HCK, HCN1, HCN4, HCRTR1, HCRTR2, HDAC1, HDAC10, HDAC11, HDAC2, HDAC3, HDAC4, HDAC5, HDAC6, HDAC7, HDAC8, HDAC9, HIF1A, HMGB1, HMGB2, HMGCR, HMOX1, HNF4A, HPGDS, HPSE, HRH2, HRH3, HRH4, HSD11B1, HSD11B2, HSD17B1, HSD17B14, HSD17B2, HSD17B3, HSD3B1, HSD3B2, HSF1, HSP90AA1, HSP90AB1, HSPA1A, HSPA6, HTR1A, HTR1B, HTR1D, HTR1F, HTR2A, HTR2B, HTR2C, HTR3A, HTR3B, HTR4, HTR5A, HTR6, HTR7, IARS, ICAM1, ICMT, IDH1, IDO1, IFNA1, IGF1R, IGFBP6, IHH, IKBKB, IL10, IL12A, IL12B, IL1B, IL2, IL37, IL4, IL6, IL6ST, IL8, IMPDH2, INSR, ITGA1, ITGA2, ITGA4, ITGA5, ITGAL, ITGAV, ITGB1, ITGB2, ITGB3, ITGB7, ITK, JAK1, JAK2, JAK3, JUN, KAT2A, KAT2B, KCNA3, KCNA5, KCNE1, KCNH2, KCNJ1, KCNK3, KCNK9, KCNMA1, KCNQ1, KDM1A, KDM4A, KDM4C, KDM4D, KDM4E, KDM5A, KDM5B, KDM6B, KDR, KIF11, KISS1R, KIT, KLK1, KLK2, KMO, LAP3, LCK, LDHA, LDHB, LDLR, LIMK1, LIPE, LMNB1, LOX, LRP6, LRRK2, LTA4H, LTB4R, LYN, LYPLA2, MALT1, MAOA, MAOB, MAP2, MAP2K1, MAP3K11, MAP3K12, MAP3K14, MAP3K5, MAP3K8, MAP4K4, MAPK1, MAPK10, MAPK14, MAPK3, MAPK8, MAPK9, MAPKAPK2, MAPT, MC1R, MC3R, MC4R, MC5R, MCHR1, MCL1, MDM2, MDP1, MERTK, MET, METAP1, METAP2, MGAM, MGLL, MIF, MIR204, MKNK2, MLC1, MLX, MME, MMP1, MMP10, MMP12, MMP13, MMP14, MMP2, MMP3, MMP7, MMP8, MMP9, MPG, MPI, MPO, MRGPRX1, MSR1, MST1R, MTAP, MTNR1A, MTNR1B, MTOR, MUSK, MYC, MYLK, NAAA, NAE1, NAMPT, NAT1, NCOA1, NCOA2, NCSTN, ND6, NEDD8-MDP1, NEK1, NEK6, NFE2L2, NFKB1, NFKBIA, NOS1, NOS2, NOS3, NOX4, NPC1L1, NPY5R, NQO1, NQO2, NR0B2, NR1H2, NR1H3, NR1H4, NR1I2, NR1I3, NR3C1, NR3C2, NR4A1, NTRK1, NTRK3, NTSR1, NUDT1, ODC1, OPRD1, OPRK1, OPRL1, OPRM1, OXER1, OXTR, P2RX3, P2RX7, P2RY1, PABPC1, PARP1, PARP2, PBK, PBRM1, PCSK7, PDE10A, PDE11A, PDE1C, PDE2A, PDE3A, PDE4A, PDE4B, PDE4D, PDE5A, PDE7A, PDE8B, PDE9A, PDF, PDGFRA, PDGFRB, PDPK1, PEPD, PER2, PFKFB3, PGD, PGGT1B, PGR, PHOSPHO1, PI4KB, PIK3CA, PIK3CB, PIK3CD, PIK3CG, PIK3R1, PIM1, PIM2, PIM3, PIN1, PKIA, PKN1, PLA2G10, PLA2G1B, PLA2G2A, PLA2G4A, PLA2G5, PLA2G7, PLAT, PLAU, PLAUR, PLD1, PLD2, PLEC, PLG, PLK1, PLK2, PNLIP, PNMT, POLA1, POLB, POLR1A, POLR2A, PON1, PORCN, PPARA, PPARD, PPARG, PPIA, PPOX, PPP1CA, PPP5C, PRC1, PRCP, PREP, PRF1, PRKACA, PRKCA, PRKCB, PRKCD, PRKCE, PRKCG, PRKCH, PRKCI, PRKCQ, PRKCZ, PRKDC, PRKG1, PRMT3, PROM1, PRSS1, PRSS3, PSEN1, PSEN2, PSENEN, PSMB1, PSMB2, PSMB5, PTAFR, PTCH1, PTEN, PTGDR, PTGDR2, PTGER1, PTGER2, PTGER3, PTGER4, PTGES, PTGS1, PTGS2, PTK2, PTK2B, PTPN1, PTPN11, PTPN2, PTPN22, PTPN3, PTPN6, PTPRC, PTPRF, PTPRS, PYGL, PYGM, QPCT, QPCTL, QRFPR, QTRT1, RAF1, RARB, RARG, RASGRP1, RASGRP3, RBBP4, RBBP7, RBP2, RBP4, RELA, REN, RET, RIPK2, RIPK3, RNASEH1, ROCK1, ROCK2, RORA, RORC, ROS1, RPS6KA1, RPS6KA3, RPS6KA5, RPS6KB1, RXRA, RXRG, S1PR1, S1PR3, SAE1, SCARB1, SCD, SCN2A, SCN5A, SCN9A, SELE, SELP, SERPINA6, SERPINE1, SHBG, SHH, SIGMAR1, SIRT1, SIRT2, SLC10A2, SLC18A3, SLC22A12, SLC22A2, SLC27A1, SLC27A4, SLC5A1, SLC5A2, SLC6A2, SLC6A3, SLC6A4, SLC6A5, SLC6A9, SLCO1B1, SMARCA4, SMN1, SMO, SNAI1, SNCA, SOAT1, SOAT2, SORD, SP1, SQLE, SRC, SRD5A1, SREBF1, SREBF2, SRF, SSTR4, ST3GAL3, ST6GAL1, STAR, STAT1, STAT3, STK17A, STK17B, STK26, STK3, STK33, STS, SUZ12, SYK, TAAR1, TACR1, TACR2, TAS2R31, TBK1, TBP, TBXA2R, TBXAS1, TDP1, TDP2, TEK, TERT, TGFBR1, TGM2, THRA, THRB, TIMP1, TK1, TKT, TLR4, TLR9, TMPRSS11D, TNF, TNFSF10, TNK2, TNKS, TNKS2, TNNC1, TNNI3, TNNT2, TOP1, TOP2A, TP53, TRAP1, TRPA1, TRPC3, TRPC6, TRPM8, TRPV1, TRPV3, TRPV4, TSPO, TTL, TTR, TUBB1, TUBB3, TWIST1, TYK2, TYMS, TYR, TYRP1, UBA2, UGCG, UGT1A1, UGT1A10, UGT1A7, UGT1A8, UGT1A9, UGT2B7, UPP1, UQCRB, UQCRFS1, UTS2R, VAV1, VCAM1, VCP, VDR, VEGFA, WEE1, WNK2, WNT1, XDH, XIAP, YAP1, YES1, ZAP70. |

**Supplementary Table S3:** Disease-Associated Targets.

| **Disease-Associated Targets** |
| --- |
| ABCA1, ABCA3, ABCB1, ABCB4, ABCC1, ABCC2, ABCC4, ABCC8, ABCG2, ACACA, ACADS, ACADVL, ACAT2, ACE, ACP1, ACSL1, ACSM3, ACTN4, ADD1, ADIPOQ, ADIPOR1, ADM, ADRA1A, ADRA1B, ADRA2A, ADRA2B, ADRA2C, ADRB1, ADRB2, ADRB3, AFP, AGER, AGT, AGTR1, AGTR2, AHR, AHSG, AIMP1, AKT1, AKT2, ALB, ALDH2, ALDH9A1, ALOX12, ALOX15, ALPP, AMBP, AMH, ANGPT1, ANGPT2, ANGPTL4, ANPEP, ANXA1, ANXA5, APOA1, APOA1-AS, APOA2, APOA5, APOB, APOC3, APOE, APOH, APOM, APP, APTX, AR, ARNT, ARSA, ASIP, ATP7B, ATRNL1, ATXN2, AZGP1, BAD, BAK1, BAX, BCL3, BDNF, BGLAP, BMP1, BMP2, BMP6, BMP7, BRAP, C3, CALCA, CALCR, CAPN10, CASP3, CASR, CAT, CAV1, CCK, CCL11, CCL2, CCL27, CCL5, CCL8, CCN2, CCND1, CCR5, CCR6, CD36, CD4, CD40, CD40LG, CD59, CD79A, CDH13, CDKN2A, CDKN2B, CDKN2B-AS1, CETP, CFD, CFL1, CFTR, CGA, CHEK2, CHGB, CHI3L1, CHKA, CHRM2, CHRM3, CLOCK, CLU, CNR1, CNR2, CNTF, COG2, COL1A1, COMT, COX5A, CP, CPB2, CPT2, CRAT, CREB1, CREBBP, CRH, CRP, CRTC2, CSF1, CSF2, CST3, CTCF, CTNNB1, CTSD, CX3CR1, CXCL11, CXCL12, CXCL5, CXCL8, CYP11A1, CYP17A1, CYP19A1, CYP21A2, CYP27A1, CYP2B6, CYP2C19, CYP2C8, CYP2C9, CYP2D6, CYP2J2, CYP3A4, CYP3A5, CYP3A7, CYP7A1, DAB2, DBP, DHCR24, DKK1, DRD1, DRD2, DRD3, DRD5, E2F1, EDN1, EGF, EHMT1, ELN, ESR1, ESR2, ESRRB, EZR, F10, F11, F13A1, F2, F3, F5, F7, F9, FABP1, FABP2, FABP3, FABP4, FADS1, FAS, FASN, FBN1, FBN2, FECH, FGB, FGF21, FGF23, FLT1, FMO3, FNDC4, FNDC5, FOS, FOXE1, FOXO1, FSTL3, FTO, GAD2, GALK1, GATA2, GATA3, GBA, GC, GCG, GCKR, GDF15, GGT1, GGT2, GH1, GHR, GHRL, GIP, GLI1, GLO1, GNB3, GNRH1, GP1BA, GPC4, GPLD1, GPT, GPX1, GRN, GSK3B, GSR, GSTM1, GSTP1, GSTT1, GTF2I, GZMB, H2AC18, HBA1, HBB, HBEGF, HCRT, HDAC2, HFE, HGF, HIF1A, HJV, HLA-A, HMGA1, HMGB1, HMGCR, HMOX1, HNF1A, HNF1B, HP, HRH1, HSD11B1, HSD11B2, HSD17B13, HSP90AA1, HSPA1B, HSPA4, HSPA5, HSPB1, HTR1B, HTR2A, HTR2C, HTR3A, IAPP, ICAM1, ID2, IFNA1, IFNA2, IFNG, IFNGR2, IFT172, IGF1, IGF1R, IGF2, IGF2R, IGFALS, IGFBP1, IGFBP2, IGFBP3, IGHE, IL10, IL12A, IL12B, IL13, IL17A, IL18, IL1A, IL1B, IL1R1, IL1RN, IL2, IL27, IL4, IL5, IL6, IL6R, IL6ST, IL7, INPPL1, INS, INSIG2, INSR, IRF1, IRF5, IRS1, IRS2, ITGAM, ITGB3, JAG1, JAK2, JUN, KCNH1, KCNJ5, KCNQ1, KISS1, KL, KLK3, KLRK1, KNG1, KRAS, KRT18, LBR, LCAT, LCN1, LCN2, LDLR, LEP, LEPQTL1, LEPR, LGALS1, LGALS3, LGALS3BP, LIMK1, LIPC, LIPE, LIPG, LMNA, LOC110386947, LOC110386951, LOX, LPA, LPL, LRP1, LRP2, LRP5, LRP6, LRP8, LTA, LTF, LYN, MACROD1, MACROD2, MAF, MAFB, MAFTRR, MAP2K1, MAP2K2, MAPK1, MAPK10, MAPK14, MAPK3, MAPK7, MAPK8, MAPK9, MB, MC4R, MEG3, MGP, MICB, MIF, MIR122, MIR126, MIR128-1, MIR128-2, MIR130A, MIR130B, MIR143, MIR144, MIR146A, MIR148A, MIR155, MIR181D, MIR186, MIR195, MIR196A2, MIR21, MIR210, MIR22, MIR223, MIR30C1, MIR34A, MIR499A, MIR509-1, MIR9-1, MIR9-3, MIR96, MKI67, MLXIPL, MME, MMP1, MMP2, MMP3, MMP7, MMP9, MPO, MSBP1, MSBP2, MSH3, MSX2, MT2A, MTHFD1L, MTHFR, MT-ND2, MTNR1B, MTOR, MTR, MTRR, MTTP, MUC1, MUC16, MYLK, NAGLU, NAMPT, NBN, NCOA2, NCOA3, NCOR1, NCOR2, NECTIN2, NEU1, NF2, NFE2L2, NFKB1, NLRP3, NM, NOD2, NOS1, NOS2, NOS3, NPPA, NPPB, NPY, NR1H2, NR1H4, NR1I2, NR1I3, NR3C1, NR3C2, NR5A2, NRF1, NRG1, NTRK2, NUP107, OLR1, OTC, OXTR, P2RX7, PAEP, PANK2, PAPPA, PAX5, PCSK1, PCSK9, PDE4D, PDE5A, PECAM1, PEX2, PF4, PGF, PGR, PGRMC1, PHACTR1, PIAS1, PIEZO1, PIK3CA, PIK3CG, PIK3R1, PLA2G10, PLA2G2A, PLA2G7, PLAT, PLAU, PLAUR, PLCG1, PLCG2, PLG, PLIN1, PLTP, PMM2, PNLIP, PNPLA3, POMC, PON1, PON2, PON3, PPARA, PPARD, PPARG, PPARGC1A, PPIG, PRKAA1, PRKAA2, PRKCA, PRKCB, PRKCD, PRKCQ, PRL, PRMT7, PTEN, PTGDR, PTGDS, PTGER3, PTGS1, PTGS2, PTH, PTHLH, PTK2B, PTPN1, PTPN22, PTTG1, PYY, RAD21, RARA, RARRES2, RBL2, RBP4, RCBTB1, RELA, REN, RETN, RFC2, RHOA, RHOD, RIPK3, ROCK1, ROCK2, RPL28P4, RUNX2, RXRG, SCARB1, SCD, SDC1, SEC14L2, SELE, SELENBP1, SELL, SELP, SERPINA12, SERPINC1, SERPINE1, SETX, SHBG, SHC1, SIRT1, SLC10A2, SLC12A2, SLC17A3, SLC17A5, SLC22A11, SLC22A12, SLC22A5, SLC22A6, SLC22A8, SLC25A37, SLC2A1, SLC2A3, SLC2A4, SLC2A9, SLC34A1, SLC8A1, SLCO1A2, SLCO1B1, SLCO1B3, SLCO2B1, SLPI, SMAD2, SMAD3, SMAD4, SOCS3, SOD1, SOD2, SOX14, SP1, SP3, SPARC, SPINK1, SPP1, SQSTM1, SREBF1, SREBF2, SST, STAP2, STAT3, STC2, STK11, SULF2, SULT1A3, SYNE2, TCF7L2, TEK, TERT, TF, TFAP2A, TFG, TFPI, TG, TGFB1, TGFBR2, TGFBR3, THBD, THBS1, THRA, TIMP1, TIMP2, TIMP3, TIMP4, TKT, TLR2, TLR4, TLR7, TMPRSS6, TNF, TNFRSF11A, TNFRSF11B, TNFRSF1B, TNFSF11, TNFSF15, TNPO3, TP53, TPD52, TPO, TRH, TRIB1, TRPS1, TRPV5, TRPV6, TSHB, TSPO, TTR, UCHL1, UCP1, UCP2, UCP3, UGT1A1, USP9X, UTS2, VAPB, VCAM1, VDR, VEGFA, VEGFB, VEGFC, VIM, VIPR1, VWF, WWOX, XBP1, XDH, XIAP, XKR6, XPA, XRCC2. |

# Supplementary Figures


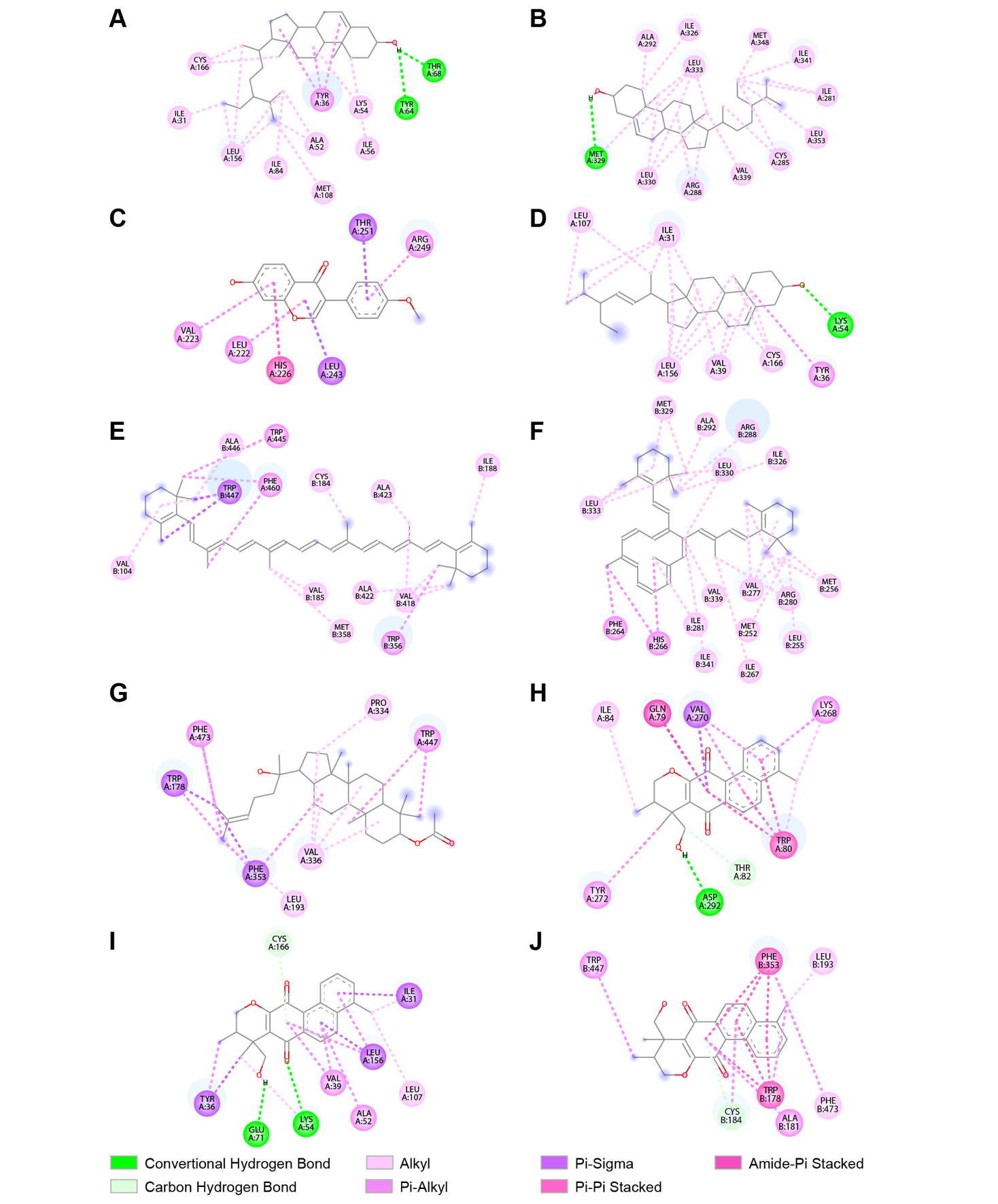


**Supplementary Figure S1.** The detailed target-compound interactions of the docking simulation. (A) MAPK1-MOL000358 interaction; (B) PPARG-MOL000358 interaction; (C) MMP9-MOL000392 interaction; (D) MAPK1-MOL000449 interaction; (E) NOS3-MOL002773 interaction; (F) PPARG-MOL002773 interaction; (J) NOS3-MOL005169 interaction; (H)AKT1-MOL007093 interaction; (I)MAPK1-MOL007093 interaction; (J) NOS3-MOL007093 interaction.


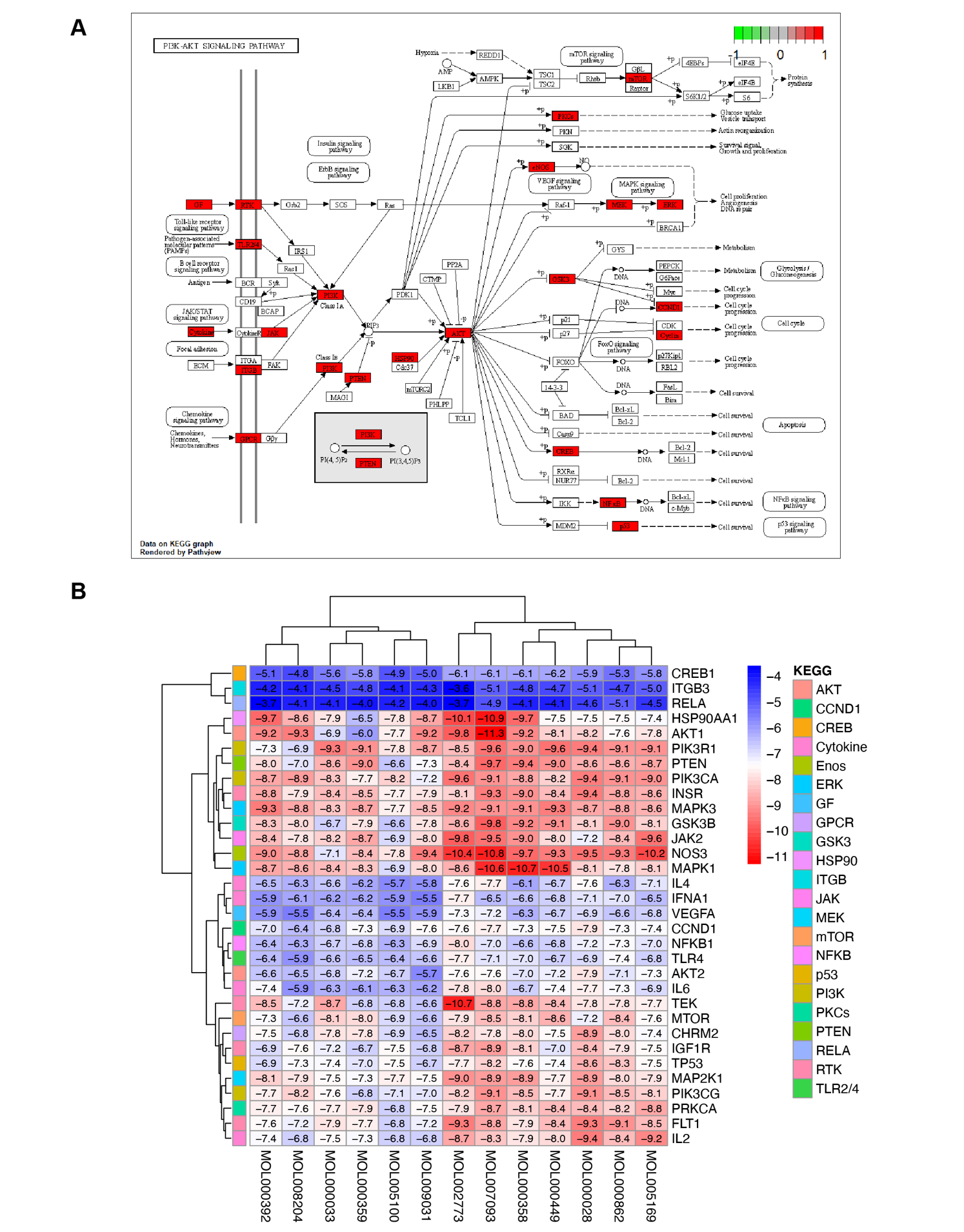


**Supplementary Figure S2.** Molecular docking analysis. (A) PI3K-AKT signaling pathway map; (B) Heatmap of binding energies.

**
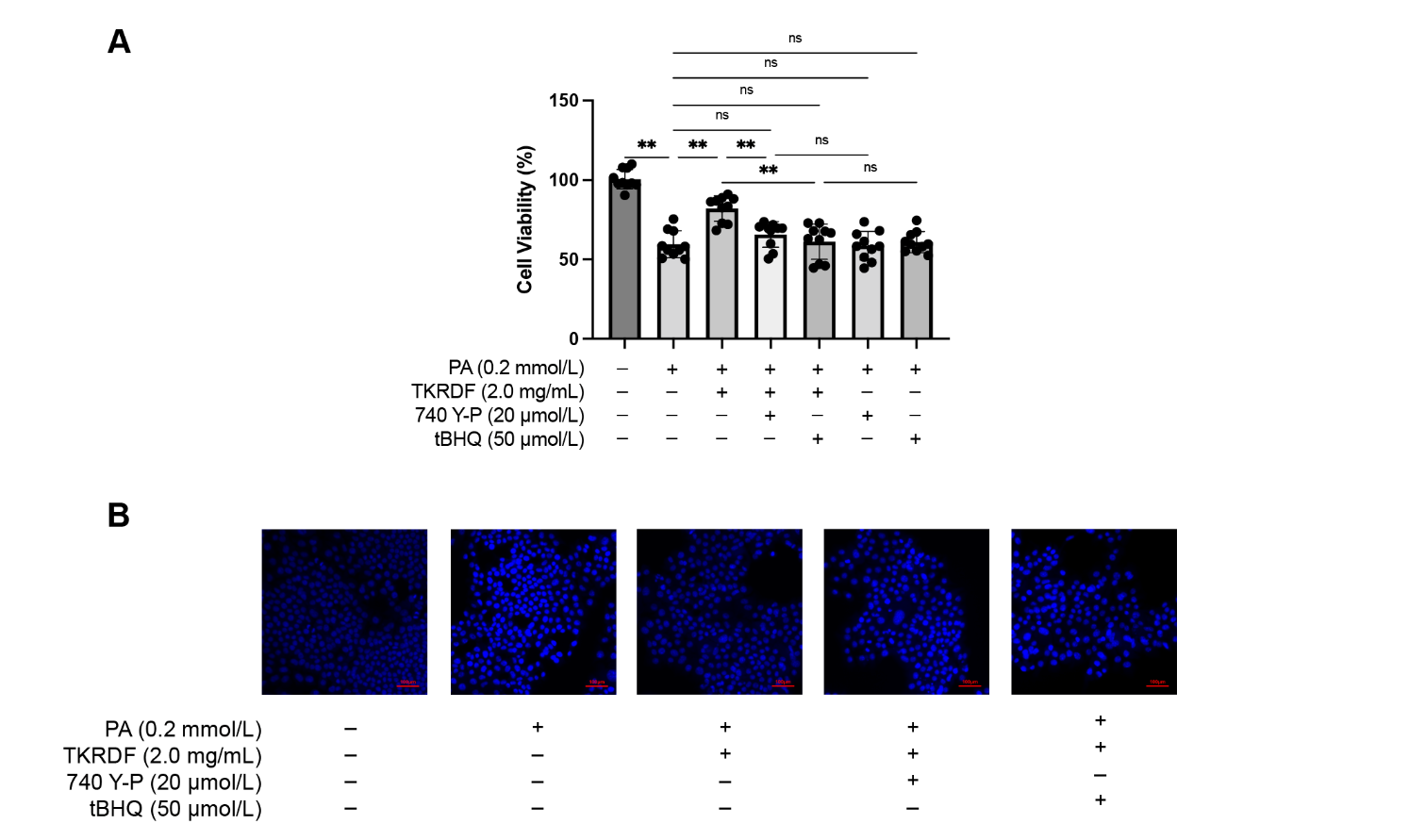
**

**Supplementary Figure S3.** PI3K agonist and ERK agonist treatment can reverse the effects of TKRDF. (A) Cell viability assay (n=10); (B) Cell apoptosis by Hoechst33258 staining. Data are means ± SD. * *p* < 0.05; ** *p* < 0.01.
